# Potential use of essential oils from *Origanum vulgare* and *Syzygium aromaticum* to control *Tetranychus urticae* Koch (Acari: Tetranychidae) on two host plant species

**DOI:** 10.7717/peerj.14475

**Published:** 2023-01-20

**Authors:** Hilal Susurluk

**Affiliations:** Faculty of Agriculture, Department of Plant Protection, Bursa Uludag University, Bursa, Türkiye

**Keywords:** Spider mite, Oregano, Clove, Toxicity, Bean, Tomato

## Abstract

Plant extracts and essential oils are considered alternatives to synthetic chemicals with toxic effects on insects and mites. Acaricidal, repellent, and oviposition effects of commercially available essential oils of *Origanum vulgare* L. (Lamiaceae) and *Syzygium aromaticum* (L.) (Myrtaceae) were investigated in this study on *Tetranychus urticae* Koch (Acari: Tetranychidae), one of the main pests in agriculture, on two host plant species using leaf disc bioassays. *O. vulgare* essential oil showed higher toxicity to *T. urticae* protonymphs and adult females inhabiting both bean and tomato leaves than *S. aromaticum* essential oil. The LC_50_ values of *O. vulgare* essential oil were found to be 1.67 and 2.05 µl L^−1^ air for the bean populations in protonymphs and adult females and 1.87 and 3.07 µl L^−1^ air for the tomato populations, respectively. Five percent concentration of *S. aromaticum* essential oil had the highest repellent effect on the bean population of *T. urticae* after 1, 24, and 48 h, resulting in 61.22%, 40.81%, and 18% repellence, respectively. Although *O. vulgare* showed higher toxic effects, *S. aromaticum* was a better repellent against the bean population of *T. urticae*. The mortality rates of adult females of *T. urticae* treated with either *O. vulgare* or *S. aromaticum* essential oil increased with the increasing concentration and time on both host plants. Both essential oils caused a decrease in egg number and larvae hatching in both bean and tomato populations of *T. urticae*. In the light of the results obtained, *O. vulgare* and *S. aromatium* essential oils have the potential to be used in the control programs against *T. urticae* in both host plants.

## Introduction

*Tetranychus urticae* Koch (Acari: Tetranychidae) is one of the main pests in agricultural fields worldwide. It has a wide host range and feeds on more than 1,100 host plant species (Santamaria et al., 2020), mainly on vegetables, fruit trees, and many industrial and ornamental plants. The direct damage caused by larvae, nymphs, and adults by sucking the plant sap can go up to the drying of the plant during periods of high population. The control of this pest is primarily based on the use of insecticides/acaricides both in Türkiye and in the world (Çağatay et al., 2018; Rincón, Rodríguez & Coy-Barrera, 2019). However, repeated applications, the overdose of these chemicals, and the high reproductive potential of the pest cause resistance to pesticides in a relatively short period. *T. urticae* has been reported to develop resistance to over 95 active compounds to date (Mota-Sanchez & Wise, 2002). As a result, there is a need for both environmentally- and human-friendly, more selective, and cost-effective control methods, which would delay *T. urticae* resistance and be an alternative to synthetic compounds. For this purpose, researchers have started to evaluate the pesticide effects of plant extracts and plant essential oils as alternatives to synthetic chemicals and their use in pest management strategies in cases of resistance (Basaid et al., 2020).

Since plants produce essential oils naturally, they have several advantages over synthetic chemicals. It has no adverse effects on the environment and human health. Essential oils do not remain in the environment permanently or have a short-term residual effect. (Isman, 2006; Ebadollahi et al., 2014). In addition, since they are complex mixtures of many active substances with different mechanisms of action, pest resistance is delayed (Pavela & Benelli, 2016). Plant essential oils can act as repellents, antifeedants, and growth regulation on insects and mites and can have lethal effects on them due to their components (Isman, 2006; Pavela & Benelli, 2016; Lengai, Muthomi & Mbega, 2020). Their toxic effects are usually achieved *via* contact, ingestion, or inhalation (Tripathi et al., 2009). Numerous studies have demonstrated that essential oils have insecticidal/acaricidal activity against spider mites, including *T. urticae* (Lee et al., 1997; Miresmailli, Bradbury & Isman, 2006; Bozhüyük, Kordalí & Kesdek, 2020). Lamiaceae, Asteraceae, Myrtaceae, and Apiaceae are the prominent plant families whose essential oils or plant extracts have been studied against spider mites (Rincón, Rodríguez & Coy-Barrera, 2019). Examples of plants that some species of *Mentha* (Labiatae), *Chrysanthemum* (Asteraceae), *Haplophyllum* (Rutaceae) (Attia et al., 2012), and *Rosmarinus officinalis* (Lamiaceae) (Miresmailli, Bradbury & Isman, 2006) have contact effect on the mite. *Lavandula angustifolia*, *Satureja hortensis*, and some *Teucrium* species (Lamiaceae) (Ebadollahi et al., 2014; Ebadollahi et al., 2015) have contact and fumigant effects. *Solanum lycopersicum* L. (Solanaceae) (Antonious & Snyder, 2006) has repellent effects on *T. urticae*. Many studies have shown that *Origanum vulgare* L. (Lamiaceae), and *Syzygium aromaticum* (L.) (Myrtaceae) Merr. & L.M. Perry was effective in the control of *T. urticae* (Choi et al., 2004; Çalmaşur, Aslan & Şahin, 2006; Eldoksch, Ayad & El-Sebae, 2009; Roh et al., 2011; Hussein, Reda & Momen, 2013; Beynaghi et al., 2015; Tabet et al., 2018; Yeşilayer & Aslan, 2018).

The pesticide effects of essential oils may be related to their multiple active components, such as monoterpene, sesquiterpene, carvacrol, and thymol (Park & Tak, 2016). Essential oil of *O. vulgare* (commonly known as oregano) consists of many monoterpenoid and monoterpene compounds with antioxidant, antimicrobial, and acaricidal effects. Its main components are carvacrol, thymol, and p-Cymene (Shang et al., 2016). The acaricidal activity of *S. aromaticum* essential oil on *T. urticae* can also be associated with its major components, eugenol and *β*-Caryophyllene (Kheradmand et al., 2015). However, variation in the chemical composition of essential oils is widespread, even within the same species (Magierowicz, Górska-Drabik & Golan, 2019). Several factors can potentially change the chemical composition and toxicity of essential oils. Some of these are the quality of the essential oil, method of extraction, plant parts used, phenological age of the plant, its geological location, and refining methods/techniques (Hussein, Reda & Momen, 2013).

It is already known that both essential oils have potential acaricidal effects on *T. urticae*. To our knowledge, these essential oils’ acaricidal and repellent effects and their impact on fertility and mortality rates have not been studied on two different host plants. Although essential oils have been used in practice for many years in pest control, very few essential oils have been commercialized in agriculture (Park & Tak, 2016). This study was conducted to reveal the potential acaricidal effect of commercially-available essential oils of *O. vulgare* and *S. aromaticum* produced in Türkiye on *T. urticae* adult females and protonymphs and to ensure the sustainability of their use. This study also focused on how the host plant factor can alter the effect of the essential oils.

## Materials & Methods

### *Tetranychus urticae* and host plants

*Tetranychus urticae* population was obtained from a strawberry greenhouse in the Aydın province of Türkiye in 2019. Moreover, the population has been grown on bean plants for about three years at Ankara University, Faculty of Agriculture, Department of Plant Protection, Ankara, Türkiye, without any pesticide application. Two separate cultures of the spider mites were grown on tomato (*Solanum lycopersicum*) and bean (*Phaseolus vulgaris*) plants. Bean and tomato plants were cultivated in plastic pots (13 cm diameter × 11 cm height) containing 350 g of growing medium consisting of perlite and peat (1:1 ratio). Irrigation was performed every other day to ensure plant growth. When the plants were fully leafed, *i.e.*, two weeks after the germination of beans and one month after the germination of tomato, plants were infested with mites, which were then allowed to adapt and reproduce on host plants for about two months. Clean bean and tomato plants for laboratory experiments and plants with mite culture were grown in separate climatized rooms at 26 ± 2 °C, 55%–60% humidity, and a photoperiod of 16 h light: 8 h dark. No solid or liquid fertilizers were used. No diseases or pests were observed in the plants during the growing period.

### Essential oils

Two essential oils were purchased from the Aksuvital Shiffa Home, 2022 (İstanbul, Türkiye) (http://www.shiffahome.com.tr). *Origanum vulgare* essential oil (İstanbul, Türkiye, Lot No:4669) was obtained from the distillation of the leaves of the plant by steam distillation method. *Syzygium aromaticum* essential oil (İstanbul, Türkiye, Lot No: 4385) was obtained from the buds of the clove plant by cold pressing method, as per the manufacturer’s instructions. The chemical compound analysis of the essential oils is shown in Table 1.

**Table 1:** Essential oils and their components.

	**Essential Oil Compounds**					**KI**	
**Peaks**	***Origanum vulgare***	**Rt**	**MC**	**MM**	***m/z***	**Ri** ^**1**^	**Ri** ^**2**^
1	*alpha*-Pinene	13,16	0.30	136	93,1	1027	1025
2	*alpha*-Thujene	13,30	0.47	136	93,1	1030	1027
3	Myrcene	19,37	0.84	136	93,1	1167	1161
4	*alpha*-Terpinene	20,22	0.58	136	121,1	1186	1178
5	*gamma*-Terpinene	23,12	2.04	136	93,1	1252	1245
6	*para*-Cymene	24,22	2.59	134	119,1	1278	1270
7	*cis*-Sabinene hydrate	31,27	0.21	154	71	1464	1460
8	Linalool	33,85	3.86	154	71	1541	1543
9	Terpinen-4-ol	35,97	0.58	154	71,1	1611	1601
10	*beta*-Caryophyllene	36,21	0.70	204	93,1	1616	1599
11	Borneol	38,95	0.86	154	95,1	1711	1700
12	*beta*-Bisabolene	39,73	1.13	204	69,1	1735	1728
13	Dipropylene glycol	42,21	30.07	134	45,1	1842	
14	Caryophyllene oxide	47,06	0.11	220	79,1	2013	1986
15	Thymol	51,16	1.33	150	135,1	2184	2164
16	Carvacrol	51,99	54.17	150	135,2	2216	2211
	***Syzygium aromaticum***						
1	*beta*-Caryophyllene	36,21	6.58	204	93,1	1616	1599
2	*alpha*-Humulene	38,42	1.80	204	93,1	1690	1667
3	Dipropylene glycol	42,20	25.44	134	45,1	1825	
4	Caryophyllene oxide	47,06	0.70	220	79,1	2013	1986
5	Eugenol	51,07	65.31	164	164	2180	2163

**Notes.**

Rt Retention Time MC Mean Composition (% Area) MM Molecular Mass *m/z* Mass to Charge Ratio KI Kovats Retention Indices Ri Retention Indices

(Ri^1^ Relative to Standard Mixture of n-alkanes in the Same Sample’s Analytical Conditions; Ri^2^ from literature).

### GC-MS analysis

The component analysis of the essential oils was determined by using the GC-MS device in the Medicinal Plants Research Laboratory of the Batı  Akdeniz Agricultural Research Institute, Antalya, Türkiye. Essential oil samples were diluted at 1:100 with hexane (99.9%, Sigma-Aldrich) and components were analyzed using a GC/GC-MS (Gas chromatography (Agilent 7890A)-mass detector (Agilent 5975C)) device and a capillary column (HP InnowaxCapillary; 60.0 m × 0.25 mm × 0.25 µm). Helium (Habaş A.Ş. Antalya, Türkiye) was used as the carrier gas (at a flow rate of 0.8 mL/min), and the samples were injected into the device as 1 µl with a split ratio of 40:1. The temperature of the injector was kept at 250 °C, and the column temperature program was set at 60 °C (10 min), from 60 °C to 220 °C at 4 °C/min and 220 °C (10 min). The total analysis time was 60 min. The mass ranged from (*m/z*) 35 to 450 and electron bombardment ionization of 70 eV was used for the mass detector, and the data of the WILEY and OIL ADAMS libraries were used to identify the components of the essential oils. The FID detector determined component percentages of the essential oils, and the component identification was performed by the MS detector (Özek et al., 2010).

### Leaf disc bioassays

#### Effects of the essential oils on adult females and protonymphs of *Tetranychus urticae*

The filter paper diffusion bioassay was conducted to determine the acaricidal effects of the tested essential oils on *T. urticae* adult females (one-to-three-day-old) and protonymphs (0-to 24-hour-old) (Choi et al., 2004). In the experiments, leaf discs of 2.0 cm in diameter were cut from clean bean or tomato leaves and placed on Petri dishes (60 mm diameter × 15 mm height). On each leaf disc, 30 adult females and/or 30 protonymphs were placed under a stereomicroscope using a fine hairy brush, and leaf discs containing mites were subsequently placed on a wet cotton pad placed on the bottom of a Petri dish. Before the experiment started, the mites were starved for approximately 3–4 h and immediately began to feed and their escape was prevented. Essential oils in various concentrations were applied directly to the Whatman No. 1 filter paper (1 cm width ×3 cm length) using an automatic pipette. After the filter papers dried for half an hour, the papers were adhered to the inner surface of Petri dishes to avoid direct contact with mites.The filter papers impregnated the essential oils were placed into the Petri dish after the mites. The Petri dishes were sealed with Parafilm (Amcor Flexibles, Zurich, Switzerland) to prevent escaping of mites. The Petri dishes were kept under a controlled atmosphere, and alive/dead counts were made after 24 h. Mites that did not move when touched with a fine brush were considered dead. The concentrations of essential oils used in the experiment were determined in preliminary studies, and 96% ethanol was used to prepare the concentrations. On the bean population, *O. vulgare* essential oil was used in 1, 2, 4, and 8 µl L ^−1^ air concentrations, and *S. aromaticum* essential oil was used in 8, 16, 24, 32, and 40 µl L ^−1^ air concentrations. On the tomato population, *O. vulgare* essential oil was applied in 1, 2, 4, 8, and 16 µl L ^−1^ air concentrations, while *S. aromaticum* essential oil was applied in 8, 16, 24, 32, 40, 80, and 160 µl L ^−1^ air concentrations. Control solutions consisted of 99.5% dipropylene glycol (Tekkim Kimya Ltd. Şti., Bursa-TR) and 96% ethanol (Tekkim Kimya Ltd. Şti., Bursa-TR) mixed in a 1:1 ratio. Since both essential oils were purchased ready-made, dipropylene glycol was present as a solvent in their contents. Triplicates were provided for each concentration and control. The spider mites that escaped during the bioassay were excluded from the experiment. No previous saturation tests have been performed on Petri dishes with essential oils.

#### Determination of repellent activity

This bioassay was carried out with modifications to the method of *Nerio, Olivero-Verbel & Stashenko (2009)* to determine the repellent effect of the tested essential oils on *T. urticae* adult females (one-to-three-day-old). To this end, plastic Petri dishes (60 mm diameter × 15 mm height), a glass spray bottle, and bean and tomato plants were used in the experiments. The two highest concentrations of *O. vulgare* and *S. aromaticum* essential oils were used for the bean and tomato populations, as significant values were not obtained at lower concentrations. These concentrations for both essential oils were 2.5% and 5% in the bean population, while 1% and 2.5% in the tomato population. The experiments were carried out in two runs. In the first application, 10.35 µl *O. vulgare* essential oil (0.5–0.7 dots/mm^2^) was sprayed on half of a bean/tomato leaf cut with a diameter of 2.0 cm, and 10.35 µl of 96% ethanol and dipropylene glycol mixture (1:1 ratio) was applied on the other half (control) using a glass spray bottle. The leaves were then left to dry for half an hour. In the second application, 10.35 µl of *S. aromaticum* essential oil (0.5–0.7 dots/mm^2^) was sprayed on half of a bean/tomato leaf cut at the same rate, and 10.35 µl of ethanol and dipropylene glycol mixture was sprayed on the other half (control). After drying for half an hour, ten adult female mites were placed on each leaf and transferred to a Petri dish with a wet cotton pad at the bottom. The lid was closed and surrounded with parafilm. The mites were counted on both halves of a leaf disc after 1, 24, and 48 h. Repellency (%) was calculated according to the formula provided below. The experiments were carried out in ten replications and repeated three times. Spraying was done at a distance of 10 cm throughout the experiment.

% *Repellency* = *(C-T/N*) ×100 (Mozaffari et al., 2013)

where *T* = number of mites (treatment), *C* = number of mites (control), *N* = number of mites (total).

#### Effects of the essential oils on the survival and reproduction of *Tetranychus urticae*

The leaf disc spray method used by *Laborda et al. (2013)* was used to evaluate the potential effects of both essential oils on the survival and reproduction of *T. urticae*. Preliminary studies were conducted to determine essential oil concentrations by diluting the oil with ethanol. These concentrations were as follows: 0.05%, 0.1%, 1%, 2.5%, and 5% (v/v) for the bean population and 0.125%, 0.25%, 0.5%, 1%, and 2.5% (v/v) for the tomato population. Ten replicates were made for each concentration. A mixture of ethanol and dipropylene glycol was used as a control. In this experiment, 20.70 µl of each concentration (0.5–0.7 dots/mm^2^) was sprayed on 2.0 cm-leaf discs of either bean or tomato and, after drying for half an hour, each leaf disc was placed in a Petri dish with a wet cotton pad. Five adult females of *T. urticae* were placed on the leaves using a fine brush. The mortality rates (%) were recorded after 1, 4, 8, 24, 72, and 120 h, and the number of eggs laid was recorded on the 1st, third, and fifth days. The numbers of emerged larvae were also scored. During this period, no phytotoxicity was observed on the leaves, and their moisture was constantly controlled.

### Statistical analyses

LC_50_ and LC_90_ values, 95% fiducial limits, and slopes of essential oils against adult females and protonymphs of *T. urticae* were calculated using the PoloPlus program (Version 2.0) (LeOra Software , 1987). The corrected mortality (%) means of adult females were transformed with the Abbott formula (Abbott, 1925) at 1, 4, 8, 24, 72, and 120 h. Along with these values, the cumulative number of eggs laid by adult females on the first, third, and fifth day, as well as the means of the cumulative number of larvae emerging, were subjected to the analysis of variance (ANOVA) (*P* < 0.05). Differences between means were evaluated according to the Tukey multiple comparison test using the JMP 13 statistical software. The mean numbers of female mites present on the treated and untreated (control) halves on tomato or bean leaf discs after 1, 24, and 48 h were compared using the *t*-test (*P* < 0.05).

## Results

### Essential oil analysis

The analysis results of the components of *O. vulgare* and *S. aromaticum* essential oils used in the study were shown in Table 1. A total of 16 compounds were identified in *O. vulgare* essential oil. The compounds above 1% were carvacrol (54.17%), dipropylene glycol (30.07%), linalool (3.86%), *para-* Cymene (2.59%), *gamma-* Terpinene (2.04%), Thymol (1.33%), and *beta-* Bisabolene (1.13%) (Table 1). Similarly, five components were found in *S. aromaticum* essential oil. These were eugenol (65.31%), dipropylene glycol (25.44%), *beta-* Caryophyllene (6.58%), *alpha-* Humulene (1.80%) and Caryophyllene oxide (0.70%), respectively (Table 1).

### Effects of the essential oils on adult females and protonymphs of *Tetranychus urticae*

The LC_50_ and LC_90_ values obtained after 24 h of exposure to *O. vulgare* and *S. aromaticum* essential oils on adult females and protonymphs of *T. urticae* in the bean and tomato populations are given in Table 2. These essential oils caused toxicity by showing a fumigant effect on adult females and protonymphs of *T. urticae*. For the bean population, LC_50_ values of *O. vulgare* essential oil applied to protonymphs and adult females were 1.67 (1.47−1.88) and 2.05 (1.82−2.29) µl L ^−1^ air, respectively, and the difference between protonymphs and adult females was not significant according to the fiducial limits. LC_50_ values of *S. aromaticum* essential oil were 16.57 (15.16–17.76) and 17.45 (15.45–19.29) µl L ^−1^ air for protonymphs and adult females, respectively, and the difference was not significant according to the fiducial limits as was the case with *O. vulgare* essential oil (Table 2). For the tomato population, LC_50_ values of *O. vulgare* essential oil applied to protonymphs and adult females were found to be 1.87 (1.62−2.13) and 3.07 (2.57−3.64) µl L ^−1^ air, respectively, while the recorded LC_50_ values of *S. aromaticum* essential oil were 22.37 (20.76–23.99) and 29.60 (27.21–32.19) µl L ^−1^ air (Table 2). *O. vulgare* essential oil showed the highest fumigant toxicity to protonymphs in the bean population with a concentration of 1.67 µl L ^−1^ air. The difference in toxicity between protonymphs and adult females in the tomato population was statistically significant for both essential oils applied. *S. aromaticum* essential oil showed lower toxicity 9.92 and 8.51-fold in bean populations, and 11.96 and 9.64-fold lower toxicity in tomato populations than *O. vulgare* essential oil on protonymphs and adult females, respectively (Table 2). *T. urticae* protonymphs in both populations treated with either essential oil were found to be more sensitive than adult females. In this experiment, the mortality rate in the control was less than 1%.

**Table 2:** Toxicity of *Origanum vulgare* and *Syzygium aromaticum* essential oils against protonymphs and adult females of the bean and tomato populations of *Tetranychus urticae* after 24 h.

**Essential oils**	**Stage**	**n** ^a^	**Slope ± SE** ^b^	**LC**_**50**_**(95% FL**^c^) [µl L^−1^ air]	**LC**_**90**_**(95% FL**^c^) [µl L^−1^ air]	*χ*^2^**(df**^d^)
**Bean population**						
*O. vulgare*	protonymphs	450	3.21 ± 0.31	1.67 (1.47–1.88)^1^	4.19 (3.57–5.21)^7^	3.23 (10)
	adult females	443	3.39 ± 0.30	2.05 (1.82–2.29)^1^	4.89 (4.18–6.01)^7^	4.83 (10)
*S. aromaticum*	protonymphs	530	7.13 ± 0.80	16.57 (15.16–17.76)^2^	25.06 (23.30–27.60)^8^	8.83 (13)
	adult females	524	6.13 ± 0.51	17.45 (15.45–19.29)^2^	28.24 (25.24–33.00)^8^	25.28 (13)
**Tomato population**						
*O. vulgare*	protonymphs	539	2.69 ± 0.24	1.87 (1.62–2.13)^3^	5.61 (4.72–7.04)^9^	5.58 (13)
	adult females	537	2.55 ± 0.20	3.07 (2.57–3.64)^4^	9.76 (7.66–13.75)^10^	19.02 (13)
*S. aromaticum*	protonymphs	530	4.67 ± 0.40	22.37 (20.76–23.99)^5^	42.05 (37.91–48.22)^11^	6.43 (13)
	adult females	678	3.51 ± 0.27	29.60 (27.21–32.19)^6^	68.53 (59.85–81.65)^12^	7.47 (18)

**Notes.**

a number of mites.

b standard error.

c fiducial limits.

d degrees of freedom.

The LC_50_ and LC_90_ values were compared for significance based on fiducial limits, labeled with numbers indicated as superscripts. The values indicated by the same number in each column (LC_50_ and LC_90_) were statistically the same according to the fiducial limits.

### Determination of repellent activity

The results of repellency data for *O. vulgare* and *S. aromaticum* essential oils are shown in Table 3. In the bean population of *T. urticae* at tested concentrations, *O. vulgare* essential oil showed a repellent effect of 51.83%, 31.77%, and 5.33% at 5% concentration. In comparison, the same oil showed a repellent effect of 40.20%, 16%, and −7.3%, at the concentration of 2.5%, after 1, 24, and 48 h, respectively. In the same population, the repellent effects of *S. aromaticum* essential oil at 5% concentration were 61.22%, 40.81%, and 18%, and was 58.90%, 38.35%, and 15.82% at 2.5% concentration, after 1, 24, and 48 h, respectively. In the bean population, both oils provided different percentages of repellency to female mites of *T. urticae* for 48 h, which decreased with time. *S. aromaticum* essential oil showed a higher repellent activity in the bean population, as compared to *O. vulgare* essential oil at both concentrations (Table 3). In the tomato population, repellence activity of *O. vulgare* essential oil was again measured after 1, 24, and 48 h and it was 0%, −20.83%, and −15.95% at 2.5% concentration, and 10.34%, −10.41%, and −3.80% at 1% concentration, respectively. In the same population, the repellent effects of *S. aromaticum* essential oil at 2.5% concentration were −27.27%, −47.47%, and −41.09%, respectively, while it was −37.27%, −38.00%, and −32.98% at 1% concentration, after same time intervals, respectively (Table 3). Except for the repellent activity obtained after 1 h at 1% concentration of *O. vulgare* essential oil in the tomato population, both essential oils did not show any repellent effect in either concentration in contrast to the bean population (Table 3).

**Table 3:** Repellent effect (%) (1, 24, and 48 h after treatment) of *Origanum vulgare* and *Syzygium aromaticum* essential oils on adult females of *Tetranychus urticae* in tomato and bean populations.

Essential oil	Populations	Concentration (%)	Repellency (%) and means of mites on the host leaves after exposure time (1, 24, and 48 h)														
			1 h					24 h					48 h				
			untreated	treated	t ratio	*P*-value	repellency	untreated	Treated	t ratio	*P*-value	repellency	untreated	treated	t ratio	*P*-value	repellency
*Origanum vulgare*	Bean	5	7.56 ± 0.14	2.40 ± 0.14	−24.53	<0001^∗^	51.83	6.56 ± 0.14	3.40 ± 0.14	−15.04	<0001^∗^	31.77	5.26 ± 0.18	4.73 ± 0.18	−2.03	=0.04^∗^	5.33
		2.5	6.80 ± 0.20	2.90 ± 0.17	−14.66	<0001^∗^	40.20	5.80 ± 0.20	4.20 ± 0.20	−5.65	<0001^∗^	16	4.63 ± 0.21	5.36 ± 0.21	2.45	=0.01^∗^	−7.3
	Tomato	2.5	4.80 ± 0.21	4.80 ± 0.27	0	=0.50	0	3.80 ± 0.22	5.80 ± 0.27	5.74	<0001^∗^	−20.83	4.13 ± 0.20	5.63 ± 0.22	4.99	<0001^∗^	−15.95
		1	5.33 ± 0.31	4.33 ± 0.35	−2.12	=0.03^∗^	10.34	4.30 ± 0.31	5.30 ± 0.35	2.11	=0.03^∗^	−10.41	4.63 ± 0.29	5.00 ± 0.33	0.82	=0.41	−3.80
*Syzygium aromaticum*	Bean	5	7.90 ± 0.18	1.90 ± 0.13	−25.70	<0001^∗^	61.22	6.90 ± 0.18	2.90 ± 0.13	−17.13	<0001^∗^	40.81	5.90 ± 0.18	4.10 ± 0.18	−25.70	<0001^∗^	18
		2.5	7.73 ± 0.15	2.00 ± 0.18	−23.94	<0001^∗^	58.90	6.73 ± 0.15	3.00 ± 0.18	−15.59	<0001^∗^	38.35	5.73 ± 0.15	4.16 ± 0.16	−6.96	<0001^∗^	15.82
	Tomato	2.5	3.60 ± 0.26	6.30 ± 0.26	7.22	<0001^∗^	−27.27	2.60 ± 0.26	7.30 ± 0.26	12.57	<0001^∗^	−47.47	2.86 ± 0.19	6.87 ± 0.19	14.42	<0001^∗^	−41.09
		1	2.30 ± 0.20	5.03 ± 0.13	11.25	<0001^∗^	−37.27	3.10 ± 0.17	6.90 ± 0.17	15.33	<0001^∗^	−38.00	3.33 ± 0.25	6.60 ± 0.27	8.65	<0001^∗^	−32.98

**Notes.**

* Significantly different between treated and untreated by *t*-test (mean ± SE, *P* < 0.05); SE means the standard error.

### Effects of the essential oils on survival and reproduction of *Tetranychus urticae*

The mean mortality rates (%) of two essential oils at different concentrations in both *T. urticae* populations after 1, 4, 8, 24, 72, and 120 h are shown in Tables 4–5. In the bean population of *T. urticae*, mortality rates after applying both essential oils increased as the applied concentration and time increased (Table 4). *O. vulgare* essential oil caused a death rate of 31.66% after 1 h at 5% concentration in the bean population, while mortality rates gradually increased to 100% at 72 h. Mortality rates at the end of 120 h were statistically significant between the lowest (0.05%) and the highest concentration (5%) applied and ranged from 19.60% to 100%, being significantly higher compared to the control (*F* = 1,341.20; *df* = 5, 54; *P* < 0001) (Table 4). Although no mortality was observed in the first 24 h in the control group, 4.90% and 8.10% mortality rates were observed after 72 and 120 h, respectively. When *S. aromaticum* essential oil was applied to the bean population at the same concentrations, higher mortality rates were observed after one hour at concentrations of 0.10%, 1%, 2.5%, and 5% compared to *O. vulgare* essential oil (Table 4). Moreover, after 120 h at the same concentrations, mortality rates varied from 17.60% to 100% and were significantly higher for *S. aromaticum* essential oil compared to control (*F* = 1,152.72; *df* = 5, 54; *P* < 0001) (Table 4). The mean number of eggs laid on leaf discs on the 1st, third , and fifth day in both populations of *T. urticae* after the application of essential oils and the mean number of larvae hatched are given in Table 6. The toxicity of *O. vulgare* and *S. aromaticum* essential oils also reduced the number of F_1_ progeny (Table 6). The egg-laying function of *T. urticae* decreased with increasing concentrations of both essential oils. While there was no death on the first day at 0.1%, 1%, and 2.5% concentrations of *O. vulgare* in the bean population (Table 4), approximately 2.15, 3.69, and 5.05 times fewer eggs laid on the fifth day in the same group, compared to the control. The progeny was inhibited due to 100% deaths on the third and fifth days at the concentration of 5% (Table 6). At 5% and 2.5% concentrations of *S. aromaticum* essential oil in the same population, the number of eggs laid by adult females on the 1st, third, and fifth day was not statistically significant. However, the difference was significant compared to the control (Table 6). Compared with the lowest concentration (0.1%), the number of larvae emerging in control was statistically significant and approximately 2.55 and 2.09 times lower in *O. vulgare* and *S. aromaticum*, respectively (Table 6).

**Table 4:** Mean mortality rates of *Origanum vulgare* and *Syzygium aromaticum* essential oils on the bean population of *Tetranychus urticae* adult females after 1, 4, 8, 24, 72, and 120 h.

Treatment % essential oil (v/v)		Mean mortality (%) after					
Bean population		1 h	4 h	8 h	24 h	72 h	120 h
*O. vulgare* essential oil							
		0 ± 0.00 b	0 ± 0.00 e	0 ± 0.00 e	0 ± 0.00 e	4.90 ± 1.26 e	8.10 ± 1.46 e
	0.05	0 ± 0.00 b	0 ± 0.00 e	0 ± 0.00 e	0 ± 0.00 e	7.90 ± 0.67 e	19.60 ± 0.61 d
	0.10	0 ± 0.00 b	9.1 ± 0.69 d	12.50 ± 0.71 d	17.36 ± 1.88 d	29.99 ± 1.44 d	59.00 ± 1.39 c
	1	0 ± 0.00 b	19.30 ± 0.55 c	29.12 ± 3.08 c	36.23 ± 4.65 c	55.10 ± 1.26 c	87.30 ± 1.28 b
	2.5	0 ± 0.00 b	43.20 ± 2.71 b	54.90 ± 1.51 b	70.90 ± 1.05 b	86.40 ± 0.96 b	90.50 ± 0.83 b
	5	31.66 ± 2.11a	68.80 ± 2.30 a	85.40 ± 1.95 a	88.37 ± 2.00 a	100.00 ± 0.00 a	100.00 ± 0.00 a
	df	5, 54	5, 54	5, 54	5, 54	5, 54	5, 54
	F	224.62	335.29	430.16	273.02	1,444.69	1,341.20
	P	<0001	<0001	<0001	<0001	<0001	<0001
*S.aromaticum* essential oil							
		0 ± 0.00 d	0 ± 0.00 d	0 ± 0.00 e	0 ± 0.00 e	10.50 ± 0.42 e	10.70 ± 0.39 f
	0.05	0 ± 0.00 d	0 ± 0.00 d	0 ± 0.00 e	0 ± 0.00 e	13.10 ± 0.69 e	17.60 ± 1.08 e
	0.10	8.26 ± 0.77 c	9.60 ± 0.81 c	10.70 ± 0.63 d	30.20 ± 0.87 d	50.90 ± 0.65 d	59.20 ± 1.47 d
	1	10.34 ± 0.64 c	12.10 ± 0.83 c	19.00 ± 1.01 c	41.30 ± 0.95 c	64.60 ± 1.15 c	73.60 ± 1.11 c
	2.5	20.80 ± 0.61 b	24.40 ± 0.88 b	33.20 ± 1.21 b	53.50 ± 1.05 b	70.50 ± 1.68 b	82.80 ± 1.41 b
	5	35.71 ± 1.10 a	57.60 ± 1.72 a	69.80 ± 2.60 a	88.32 ± 1.42 a	92.06 ± 1.21 a	100.00 ± 0.00 a
	df	5, 54	5, 54	5, 54	5, 54	5, 54	5, 54
	F	435.43	571.2	434.68	1,416.51	950.51	1,152.72
	P	<0001	<0001	<0001	<0001	<0001	<0001

**Notes.**

Means in columns followed by the same letters are not significantly different (Tukey-Kramer Test, *P* > 0.05) df = degrees of freedom.

**Table 5:** Mean mortality rates of *Origanum vulgare* and *Syzygium aromaticum* essential oils on the tomato population of *Tetranychus urticae* adult females after 1, 4, 8, 24, 72, and 120 h.

Treatment % essential oil (v/v)		Mean mortality (%) after					
Tomato population		1 h	4 h	8 h	24 h	72 h	120 h
*O. vulgare* essential oil							
	Control	0 ± 0.00 c	0 ± 0.00 c	0 ± 0.00 c	0 ± 0.00 f	6.10 ± 1.44 f	8.60 ± 1.43 e
	0.125	0 ± 0.00 c	0 ± 0.00 c	0 ± 0.00 c	26.10 ± 0.48 e	38.70 ± 0.66 e	65.60 ± 1.46 d
	0.25	0 ± 0.00 c	0 ± 0.00 c	0 ± 0.00 c	31.40 ± 0.33 d	43.80 ± 0.62 d	70.90 ± 0.52 c
	0.5	0 ± 0.00 c	0 ± 0.00 c	0 ± 0.00 c	40.10 ± 0.73 c	54.80 ± 1.30 c	78.20 ± 0.86 b
	1	3.20 ± 0.29 b	8.90 ± 0.27 b	14.50 ± 0.65 b	50.60 ± 0.77 b	69.00 ± 0.39 b	100.00 ± 0.83 a
	2.5	7.60 ± 0.65 a	13.40 ± 0.40 a	38.10 ± 0.52 a	62.60 ± 1.15 a	74.90 ± 0.50 a	100.00 ± 0.00 a
	df	5, 54	5, 54	5, 54	5, 54	5, 54	5, 54
	F	114.01	891.82	2,045.41	994.52	731.88	1,295.99
	P	<0001	<0001	<0001	<0001	<0001	<0001
*S.aromaticum* essential oil							
	Control	0 ± 0.00 c	0 ± 0.00 d	0 ± 0.00 e	0 ± 0.00 f	10.10 ± 0.98 e	10.90 ± 1.10 f
	0.125	0 ± 0.00 c	0 ± 0.00 d	0 ± 0.00 e	12.70 ± 0.81 e	31.20 ± 0.62 d	34.80 ± 0.77 e
	0.25	0 ± 0.00 c	0 ± 0.00 d	7.50 ± 0.65 d	22.30 ± 0.80 d	52.40 ± 0.93 c	60.20 ± 0.66 d
	0.5	0 ± 0.00 c	8.70 ± 0.47 c	12.20 ± 0.71 c	32.60 ± 1.55 c	52.40 ± 0.74 c	72.30 ± 1.34 c
	1	5.00 ± 0.21 b	22.20 ± 0.72 b	34.40 ± 1.19 b	55.20 ± 1.06 b	77.40 ± 1.30 b	86.30 ± 0.78 b
	2.5	9.10 ± 0.48 a	31.10 ± 0.87 a	56.50 ± 1.38 a	80.40 ± 1.92 a	100.00 ± 0.00 a	100.00 ± 0.00 a
	df	5, 54	5, 54	5, 54	5, 54	5, 54	5, 54
	F	323.89	707.67	713.07	608.19	1,361.10	1,409.54
	P	<0001	<0001	<0001	<0001	<0001	<0001

**Notes.**

Means in columns followed by the same letters are not significantly different (Tukey–Kramer Test, *P* > 0.05) df*=* degrees of freedom.

**Table 6:** Means the number of eggs laid by *Tetranychus urticae* females after the 1st, 3rd, and 5th day and the number of emerged larvae in the bean and tomato populations.

Treatment % essential oil (v/v)					
Bean population	Means of the total number of eggs laid				
*O. vulgare* essential oil		1 day	3 days	5 days	Means of emerging larvae
	Control	13.00 ± 1.26 a	25.16 ± 2.15 a	34.50 ± 2.17 a	32.33 ± 1.89 a
	0.1	4.80 ± 0.65 b	10.83 ± 0.94 b	16.00 ± 1.21 b	12.67 ± 1.67 b
	1	2.83 ± 0.30 bc	6.00 ± 0.73 c	9.33 ± 0.66 c	6.00 ± 1.03 c
	2.5	2.33 ± 0.33 bc	4.16 ± 0.40 cd	6.83 ± 0.94 cd	4.33 ± 1.08 cd
	5	1.00 ± 0.44 c	0.00 ± 0.00 d	0.00 ± 0.00 d	0.00 ± 0.00 d
	df	4, 25	4, 25	4, 25	4, 25
	F	47.07	75.96	84.17	94.23
	P	<0001	<0001	<0001	<0001
*S.aromaticum* essential oil	Control	16.16 ± 1.30 a	29.80 ± 0.60 a	35.67 ± 1.74 a	32.50 ± 1.78 a
	0.1	15.16 ± 1.13 ab	18.83 ± 1.07 b	23.50 ± 0.71 b	15.50 ± 1.38 b
	1	11.33 ± 0.95 bc	15.00 ± 0.68 c	19.50 ± 0.76 b	14.50 ± 1.38 b
	2.5	10.00 ± 0.85 cd	10.00 ± 0.85 d	11.33 ± 1.11 c	7.16 ± 1.13 c
	5	6.50 ± 0.61 d	7.00 ± 0.36 d	8.00 ± 0.51 c	4.50 ± 0.34 c
	df	4, 25	4, 25	4, 25	4, 25
	F	15.43	138.89	105.26	70.92
	P	<0001	<0001	<0001	<0001
Treatment % essential oil (v/v)					
Tomato population	Means of the total number of eggs laid				
*O. vulgare* essential oil		1 day	3 days	5 days	Means of emerging larvae
	Control	14.00 ± 0.63 a	25.50 ± 2.23 a	32.83 ± 2.93 a	29.83 ± 2.85 a
	0.25	11.50 ± 0.67 b	15.16 ± 0.47 b	20.50 ± 0.56 b	16.50 ± 1.36 b
	0.5	8.16 ± 0.30 c	11.00 ± 0.36 b	14.50 ± 0.56 c	10.00 ± 0.93 c
	1	2.50 ± 0.22 d	2.50 ± 0.22 c	2.00 ± 0.25 d	0.50 ± 0.22 d
	2.5	1.00 ± 0.25 d	1.00 ± 0.25 c	0.66 ± 0.33 d	0.00 ± 0.00 d
	df	4, 25	4, 25	4, 25	4, 25
	F	148.33	91.52	95.20	70.52
	P	<0001	<0001	<0001	<0001
*S.aromaticum* essential oil	Control	15.00 ± 0.47 a	28.00 ± 1.75 a	37.00 ± 2.93 a	33.16 ± 2.66 a
	0.25	13.17 ± 0.67 b	16.50 ± 0.42 b	22.00 ± 0.57 b	18.00 ± 0.93 b
	0.5	3.00 ± 0.00 c	6.17 ± 0.60 c	7.83 ± 0.79 c	4.00 ± 0.51 c
	1	1.50 ± 0.20 d	2.67 ± 0.21 cd	2.83 ± 0.16 cd	2.00 ± 0.25 c
	2.5	0.50 ± 0.20 d	0.80 ± 0.17 d	0.00 ± 0.00 d	0.00 ± 0.00 c
	df	4, 25	4, 25	4, 25	4, 25
	F	601.65	174.86	124.06	119.11
	P	<0001	<0001	<0001	<0001

**Notes.**

Means in each column followed by the same letters are not significantly different (Tukey-Kramer Test, *P* > 0.05) df = degrees of freedom.

In the tomato population of *T. urticae*, mortality rates for both essential oils also increased over time depending on the concentration (Table 5). *O. vulgare* essential oil caused death rates in the first 1 h at 2.5% and 1% concentrations, and 100% mortality was observed after 120 h at these concentrations. Additionally, the mortality rates at the end of 120 h were significantly different at the lowest (0.125%) and the highest concentration (2.5%), being 65.60% and 100% (*F* = 1,295.99; *df* = 5, 54; *P* < 0001), respectively. The rates also showed statistical significance for *S. aromaticum* essential oil (*F* = 1,409.54; *df* = 5, 54; *P* < 0001), and the values between 34.80% and 100% were recorded (Table 5). When *O. vulgare* essential oil was applied to the tomato population of mites at concentrations as low as 0.125%, 0.25%, and 0.5%, the mortality rates in the first 8 h were not statistically significant compared to the control. From the 24th hour, significantly different mortality rates were recorded at the 0.125% concentration, compared to the control (Table 5). When *S. aromaticum* essential oil was applied to the tomato population at low concentrations (0.125% and 0.25%), mortality rates in the first four hours were not significantly different compared to control, while the percentage mortality at 0.25% concentration was seen after 8 h (Table 5). While no mortality rate was observed in the first 24 h in the control group for both essential oils in the tomato population, a mortality rate close to 10% was observed at the end of 120 h. Significant differences between the control and any concentration of *O. vulgare* and *S. aromaticum* essential oils were recorded in the tomato population on the 1st, third, and fifth days. The number of eggs laid by *T. urticae* was approximately 1.60 and 1.68 times less at the concentration of 0.25% on the fifth day of *O. vulgare* and *S. aromaticum*, respectively (Table 6). No larvae emerged at the highest concentration (2.5%) for both essential oils, and a 1.80- and 1.84-fold decrease were observed in the number of larvae at the lowest concentration (0.25%) of *O. vulgare* and *S. aromaticum*, respectively, compared with control treatment.

## Discussion

The present study demonstrated that essential oils from *O. vulgare* and *S. aromaticum* had an acaricidal effect on adult females and protonymphs of *T. urticae* on bean and tomato plants, although the characteristics of this effect varied. Both oils’ fumigant toxicity and repellent effect were higher in the bean population of *T.urticae*. However, earlier death and a decrease in the number of eggs laid in the tomato population were observed at 2.5% concentrations. In contrast, the same effect was achieved with double that amount in the bean population. These results suggest that *O. vulgare* and *S. aromaticum* essential oils can potentially be used in the control strategies against *T. urticae*.

The biological activities of plant essential oils are related to the monoterpenes and phenols in their contents (Amizadeh, Hejazi & Saryazdi, 2013). Consistent with previous studies (Singh, Baghotia & Goel, 2012; Koc et al., 2013; Kheradmand et al., 2015; Imtara et al., 2021), the main component of *O. vulgare* essential oil used in this study was carvacrol (54.17), while the main component of *S. aromaticum* essential oil was eugenol (65.31) (Table 1). In previous studies, different substances have also been shown as the main component in *O. vulgare* essential oil. For example, Onaran et al. (2014) reported mainly thymol (50.41%) and carvacrol (12.96%) in the chemical composition of *O. vulgare* essential oil, while Şahin et al. (2004) found caryophyllene and spathulenol to be major components. Generally, the higher percentage of carvacrol in the essential oil is associated with a stronger acaricidal or insecticidal effect (Koc et al., 2013). In the present study, *O. vulgare* essential oil showed very high toxicity to protonymphs and adult females of *T. urticae* in both bean and tomato populations within 24 h. Specifically, 100% mortality was observed at 5% concentration in the bean population and 2.5% in the tomato population. Consistent with the present study, Miresmailli, Bradbury & Isman (2006) found that some individual compounds of *Rosmarinus officinalis* essential oil, such as borneol and bornyl acetate, were not toxic to mites feeding on bean plants were relatively toxic to mites feeding on tomato plants. In this study, the differences in the bean and tomato populations after the experiment may be due to some chemical components found in both essential oils. Pavela et al. (2016) reported that the chemical composition of essential oils significantly affects fumigation toxicity in all developmental stages of *T. urticae*. In similar studies, natural monoterpenes such as linalool, carvone, and menthol (Badawy, El-Arami & Abdelgaleil, 2010), and carvacrol and thymol (Pavela & Benelli, 2016) have a potent fumigant effect against all stages of *T. urticae*. Therefore, the toxic effects of *O. vulgare* essential oil reported in this study may result from the synergistic effect of several compounds. Eugenol is the main compound responsible for clove’s antioxidant, antifungal, antiviral, and insecticidal activities, making it a medically important drug. The percentage of eugenol in *S. aromaticum* essential oil used in this study was 65.31%, and it was found to be less effective against protonymphs and adults of *T. urticae* than *O. vulgare* in both host plants. Similarly, *Eldoksch, Ayad & El-Sebae (2009)* reported that the phenol compound eugenol has an acaricidal effect on *T. urticae*. This effect may be due to a phenolic function that can increase the acaricidal properties of terpenes. This finding was supported by Hussein, Reda & Momen (2013), who found *S. aromaticum* (syn. *Eugenia caryophyllata*) essential oil to be less toxic than *Triticum vulgare* and *Eucalyptus globulus* essential oils in adult females of *T. urticae.* Conversely, Kheradmand et al. (2015) and Beynaghi et al. (2015) reported a lower LC_50_ value (6.13 µl L ^−1^ air) of *S. aromaticum* essential oil in *T. urticae* adults on bean leaf discs after 24 h compared to the present study (LC_50_ = 17.45 µl L ^−1^ air). This result may be due to the difference in the chemical composition of *S. aromaticum* essential oil. While the percentages of eugenol, *β*-Caryophyllene and *α*-Humulene in *S. aromaticum* essential oil used in the study of Kheradmand et al. (2015) were 78.5%, 13.8%, and 2.8%, in this study, these values were 65.31%, 6.58%, and 1.80%, respectively.

The Lamiaceae family has excellent potential in pest management strategies due to having both insecticide and acaricide properties (Ebadollahi, Ziaee & Palla, 2020). The *Origanum* genus from this family has been investigated for these effects extensively. For example, *O. vulgare* essential oil has been demonstrated to have a very high acaricidal effect on adult females of *T. urticae* at a concentration of 8.52 mg L^−1^ air (Mahmoud et al., 2019), while its hydroethanolic extract caused 75% of mortality in *T. urticae* adult females after 24 h in another report (Tabet et al., 2018). The essential oil vapors of *O. vulgare* at a concentration of 2 µl L ^−1^ air caused a mortality rate of 95% in adults and nymphs of *T. urticae* in 120 h (Çalmaşur, Aslan & Şahin, 2006). In contrast, its vegetable oil achieved a 100% mortality rate at 19 × 10^−3^ µl L ^−1^ air concentrations after 24 h on *T. urticae* adults (Choi et al., 2004). In another study, essential oil from *O. onites* containing 68.23% carvacrol showed a dose-dependent acaricidal effect against *Tetranychus cinnabarinus* (Sertkaya, Kaya & Soylu, 2010). The reports focusing on the toxicity of plants in the Myrtaceae family on *T. urticae* suggest a broad range of results (Rincón, Rodríguez & Coy-Barrera, 2019). In a previous study, *S. aromaticum* essential oil (0.1% concentration) caused a 41.3% mortality rate in *T. urticae* adult females by leaf disc residue dipping method (Roh et al., 2011). In another study, *Lee et al. (1997)* reported that carvacrol, thymol, and eugenol from monoterpenoids exhibited 100% mortality at 10.000 ppm after 24 h in *T. urticae*. Similar to the present study, *S. aromaticum* (1.5% concentration) showed a mortality rate of 53.33, 63.33, 73.33 and 98.54% in *Oligonychus coffeae* Nietner (Acari: Tetranychidae) after 24, 48, 72 and 120 h, respectively (Barua et al., 2015).

In the present study, *S. aromaticum* essential oil at 2.5% and 5% concentrations showed a more substantial repellent effect on adult females of *T. urticae* compared to *O. vulgare* in the bean population for ≈ 48 h after the application. However, the opposite was observed in tomato plants, *i.e.* , the same concentrations of the essential oil (1% and 2.5%) attracted adult females of *T. urticae* toward tomato plants. Hence, the repellent effects differed depending on the host plant and the type of essential oil applied. This finding is noteworthy because, to our knowledge, the present study appears to be the first to compare the repellent effects of two different essential oils on two different host plants in *T. urticae*. It has been reported that phenols such as eugenol have a more substantial repellent potential than monoterpenes such as *p*-cymene found in *Origanum* species (Koul, Walia & Dhaliwal, 2008). The results of this study are compatible with this line of information. Conversely, in a repellency trial on different *Origanum* species, *O. vulgare* essential oil showed a repellent effect of 70.3% at the concentration of 0.5% and 95.4% at the concentration of 4% after 48 h (Yeşilayer & Aslan, 2018). Carvacrol and thymol showed toxic and repellent activity on *T. urticae* (Tak & Isman, 2017). Kheradmand et al. (2015) reached similar results in experiments with essential oils of *S. aromaticum*, which had a substantial potential repellent effect on *T. urticae* adults. In the present study, the attractive effect of *S. aromaticum* essential oil when applied to a tomato leaf may be due to the structure of the plant, that of eugenol, or some other minor compounds present in the essential oil. Eugenol was shown to have an attractant effect on some pests such as flies (Reis et al., 2016). Repellents lose their effects quickly as they contain volatile compounds (Nerio, Olivero-Verbel & Stashenko, 2010). The high repellent effect of *S. aromaticum* oil on the bean plant in a short time is significant in terms of dispersing the *T. urticae* population, preventing egg-laying, thus reducing the F_1_ generation and eventually preventing the damage it will cause to the plant. Like this study, rosemary oil has been shown to have a repellent effect on *T. urticae* for 6 h, after which the effect declined gradually (Ebadollahi et al., 2015).

When the two essential oils were applied to the tomato population, no repellent effect was observed on the tomato plant. However, there was still a decrease in the number of eggs laid by the females; resulting in a decreased number of larvae. It could be argued that some substances synthesized by tomato plants can reduce the nutritional and reproductive performance of *T. urticae* and/or affect the toxicity of essential oils to *T. urticae*. It is still unclear which volatiles or other compounds synthesized by the tomato plant can cause such an effect; however, a possible explanation for this might be the presence of physical and chemical barriers as a resistance mechanism against *T. urticae* in wild tomato genotypes (Santamaria et al., 2020) that is decreased or absent in cultivated tomatoes. Moreover, a tomato plant is inherently more hostile to *T. urticae* as the presence of glandular hair may direct mites to fall into a trap, and some toxic substances can cause direct poisoning (Gotoh et al., 1993). It is thought that the differences observed in toxicity and repellent effects on mites and their reproduction in tomato and bean populations recorded in this study may be related to plant resistance and/or individual and mixture differences between the components in both oils. However, much more detailed studies are needed to prove this hypothesis.

As far as is known, there is no study showing the oviposition-inhibiting effect of *O. vulgare* and *S. aromaticum* essential oils on two different populations of *T. urticae*. In a recent report, the effect of a sublethal dose of *S. aromaticum* essential oil (LC_25_) on the demographic parameters of *T. urticae* has been studied, and the total number of eggs laid in the trial was found to be reduced by about half as compared to the control (Beynaghi et al., 2015). This study observed that *O. vulgare* and *S. aromaticum* essential oils significantly inhibited the number of eggs laid in both host plants and, thus, the development of F_1_ progeny. It is thought that both essential oils can directly affect fertility. Carvacrol, *para*-Cymene, *gamma*-Terpinene, and eugenol compounds, which are the major compounds of *O. vulgare* and *S. aromaticum* essential oils may have impaired reproductive behavior, individually or in combination, or both may have an effect. Roh et al. (2011) reached similar results in experiments with *Thymus vulgaris* and *O. vulgare* essential oils, which are rich in thymol and carvacrol. They identified these as potential antifeedants and oviposition inhibitors to *T. urticae*. Similarly, *O. majorana* and *O. compactum* strongly inhibited oviposition behavior by more than 80% in *T. urticae* (Pavela & Benelli, 2016), and *O. onites* essential oil inhibited the same behavior of *Planococcus citri* females by 63.7%. *Rosmarinus officinalis* and *Majorana hortensis* oils caused a significant reduction in the total numbers of eggs laid in *T. urticae* over the ten days at all concentrations tested (0.125%–2%) (Amer, Refaat & Momen, 2001). It has been reported that essential oils containing carvacrol, thymol, and anethole can be recommended as reproduction-deterring fumigants for the control of greenhouse pest *T. cinnabarinus* (Erler & Tunc, 2005). *Santamaria et al. (2020)* showed that 7-epizingiberene, a sesquiterpene found in a tomato plant, reduced mite fecundity and affected population density.

Since essential oils are complex mixtures, they have more than one mechanism of action Miresmailli, Bradbury & Isman, 2006. The fact that both oils cause high mortality in tomato and bean populations in a very short time period such as 24 h shows that these essential oils may be affecting the nervous system. Similar observations were recorded by Sendi & Ebadollahi (2013). Similarly, Plata-Rueda et al. (2021) reported that the very rapid action of *O. vulgare* essential oil on *T. molitor* (Col.: Tenebrionidae) may be due to the effect of terpenoids in the oil on the nervous system, as seen in other insects exposed to essential oils. The fact that the oils were also effective on the reproduction of *T. urticae* in this study shows that they have very different mechanisms of action. For these reasons, the effect of both oils on *T. urticae* populations should be evaluated in more detail by applying both oils in a mixture or separately under greenhouse and field conditions.

## Conclusions

*Origanum vulgare* and *Syzygium aromatium* essential oils have been quite successful in control of *T. urticae*. Both essential oils showed toxicity to *T. urticae*, and significantly reduced oviposition and *F*_1_ progeny in both host plants. Both essential oils caused 100% mortality in both host plants after 120 h. But, the host plant to which the oil was applied changed the effect of the essential oil. While the essential oils had a repellent effect in bean plant, the same effect was not observed in tomato plant. This results show that these essential oils can be studied in more detail. There is a need for more studies on each component’s role in these oils’ toxicity, such as their individual and combined effects, behavior, and mechanism of action in field or greenhouse conditions. It is thought that the results obtained will be valuable in developing alternative methods to synthetics in the control of *T. urticae*.

##  Supplemental Information

10.7717/peerj.14475/supp-1 Supplemental Information 1 Chromatogram results

10.7717/peerj.14475/supp-2 Supplemental Information 2 Percent mortality values after experiments

10.7717/peerj.14475/supp-3 Supplemental Information 3 Fecundity experiment results

10.7717/peerj.14475/supp-4 Supplemental Information 4 Gas Chromatogram Profiles of O. vulgare Gas chromatogram profiles of peak retention of constituents of *Origanum vulgare* essential oil: *alpha*-Pinene (1), *alpha*-Thujene (2), Myrcene (3), *alpha*-Terpinene (4), *gamma*-Terpinene (5), *para*-Cymene (6), *cis*-Sabinene hydrate (7), Linalool (8), Terpinene-4-ol (9), *beta*-Caryophyllene (10), Borneol (11), *beta*-Bisabolene (12), Dipropylene glycol (13), Caryophyllene oxide (14), Thymol (15), Carvacrol (16).

10.7717/peerj.14475/supp-5 Supplemental Information 5 Gas Chromatogram Profiles of S. aromaticum *beta*-Caryophyllene (1), *alpha*-Humulene (2), Dipropylene glycol (3), Caryophyllene oxide (4), Eugenol (5).

10.7717/peerj.14475/supp-6 Supplemental Information 6 Repelleny results

## Additional Information and Declarations

### Data Availability

The following information was supplied regarding data availability: The raw data are available in the Supplementary Files .
