# Supplementary material for: Potential use of essential oils from Origanum vulgare and Syzygium aromaticum to control Tetranychus urticae Koch (Acari: Tetranychidae) on two host plant species
Source: PeerJ. 2023 Jan 20;11:e14475. doi: 10.7717/peerj.14475 (PMC9869773; doi:10.7717/peerj.14475)

Fig. 1
Gas chromatogram profiles of peak retention of constituents of *Origanum vulgare* essential oil: *alpha*-Pinene (1), *alpha*-Thujene (2), Myrcene (3), *alpha*-Terpinene (4), *gamma*-Terpinene (5), *para*-Cymene (6), *cis*-Sabinene hydrate (7), Linalool (8), Terpinene-4-ol (9), *beta*-Caryophyllene (10), Borneol (11), *beta*-Bisabolene (12), Dipropylene glycol (13), Caryophyllene oxide (14), Thymol (15), Carvacrol (16).

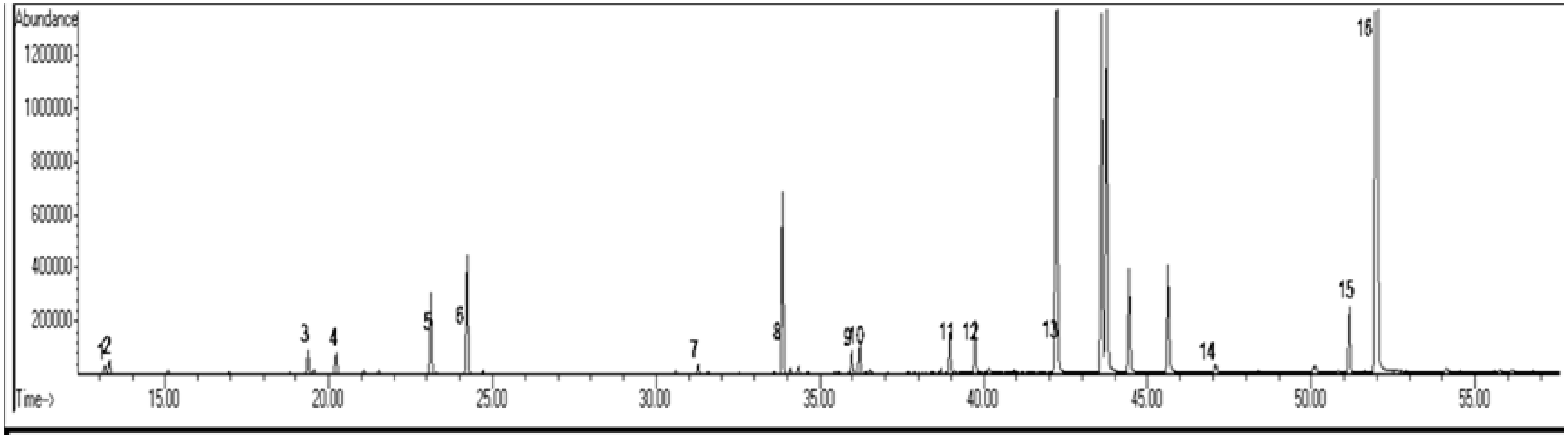

Supplement: Supplemental Information 4 — Gas chromatogram profiles of peak retention of constituents of Origanum vulgare essential oil: alpha-Pinene (1), alpha-Thujene (2), Myrcene (3), alpha-Terpinene (4), gamma-Terpinene (5), para-Cymene (6), cis-Sabinene hydrate (7), Linalool (8), Terpinene-4-ol (9), beta-Caryophyllene (10), Borneol (11), beta-Bisabolene (12), Dipropylene glycol (13), Caryophyllene oxide (14), Thymol (15), Carvacrol (16). [file peerj-11-14475-s004.pdf]
