# Supplementary material for: Potential use of essential oils from Origanum vulgare and Syzygium aromaticum to control Tetranychus urticae Koch (Acari: Tetranychidae) on two host plant species
Source: PeerJ. 2023 Jan 20;11:e14475. doi: 10.7717/peerj.14475 (PMC9869773; doi:10.7717/peerj.14475)

Fig. 2

Gas chromatogram profiles of peak retention of constituents of *Syzygium aromaticum* essential oil: *beta*-Caryophyllene (1), *alpha*-Humulene (2), Dipropylene glycol (3), Caryophyllene oxide (4), Eugenol (5).

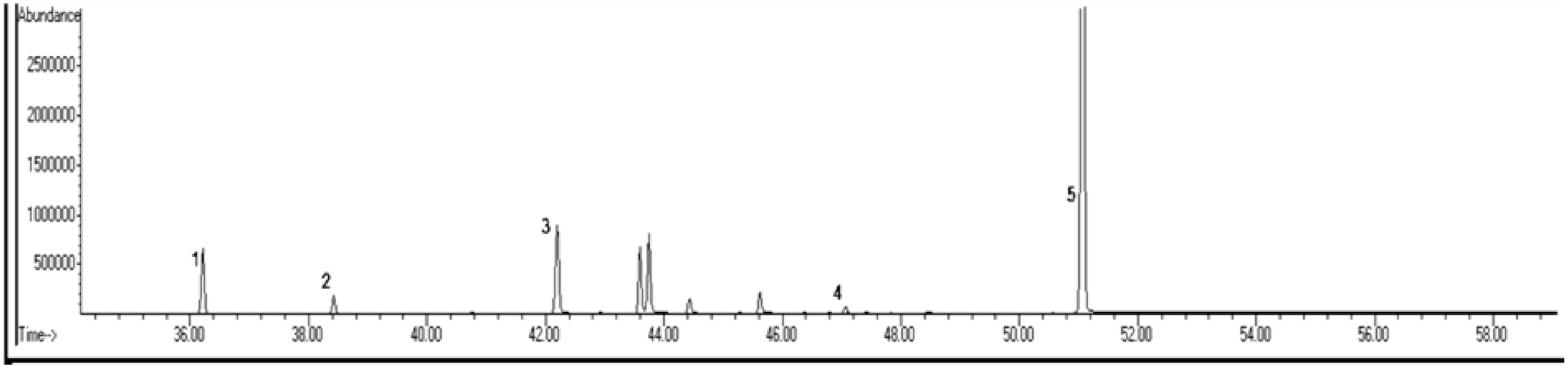

Supplement: Supplemental Information 5 — beta-Caryophyllene (1), alpha-Humulene (2), Dipropylene glycol (3), Caryophyllene oxide (4), Eugenol (5). [file peerj-11-14475-s005.pdf]
