# Supplementary figures and images for: Potential use of essential oils from Origanum vulgare and Syzygium aromaticum to control Tetranychus urticae Koch (Acari: Tetranychidae) on two host plant species
Source: PeerJ. 2023 Jan 20;11:e14475. doi: 10.7717/peerj.14475 (PMC9869773; doi:10.7717/peerj.14475)

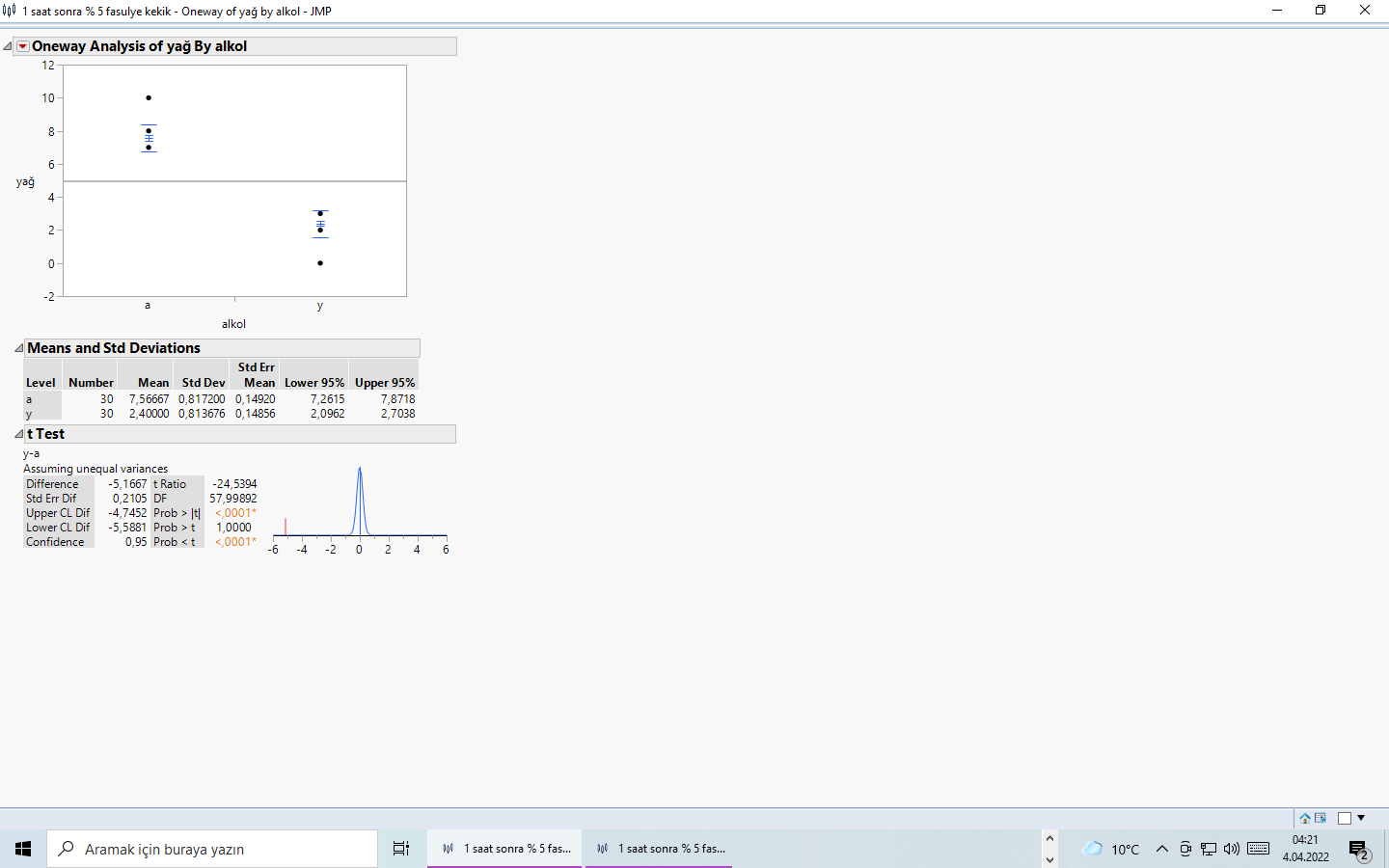


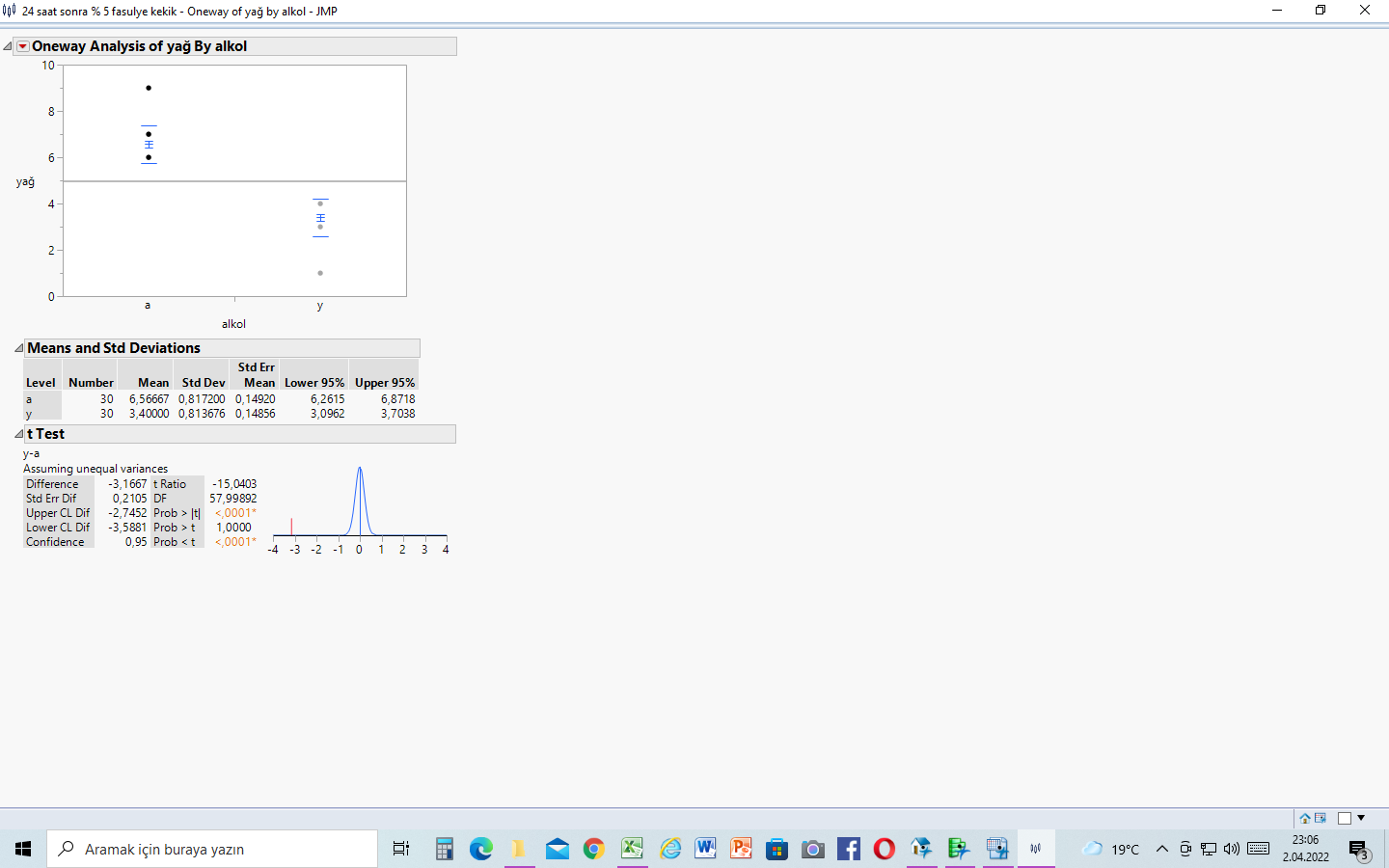


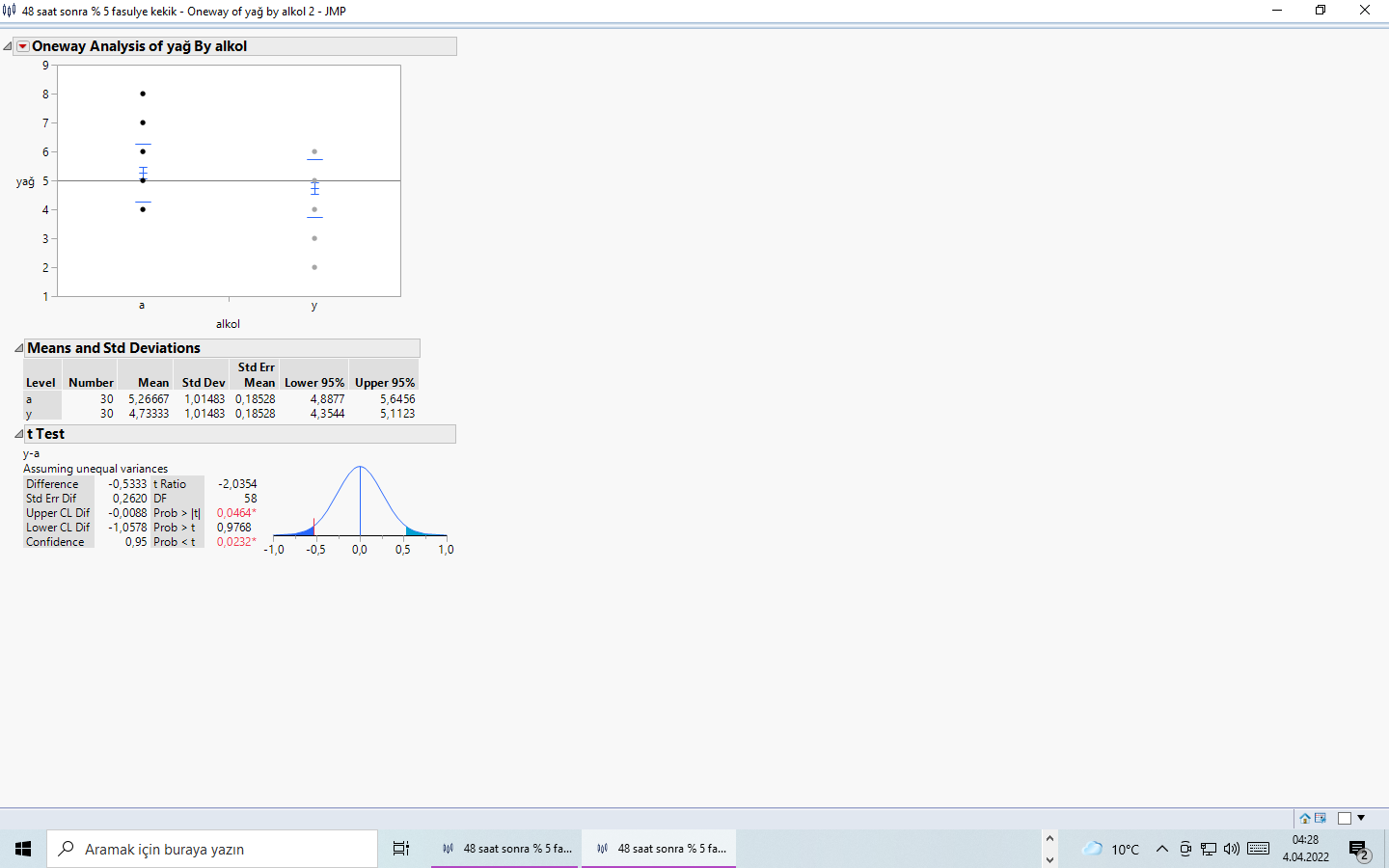


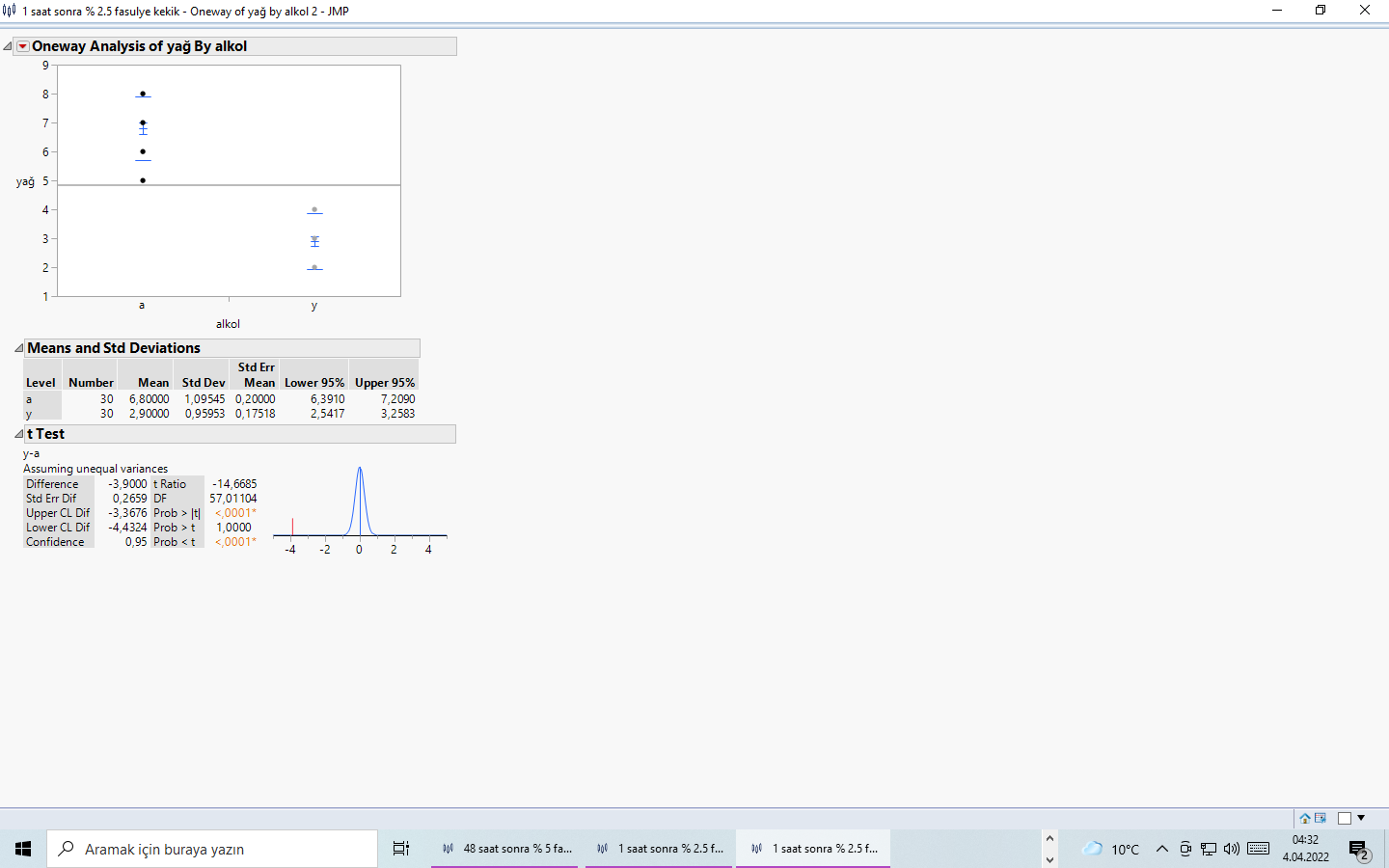


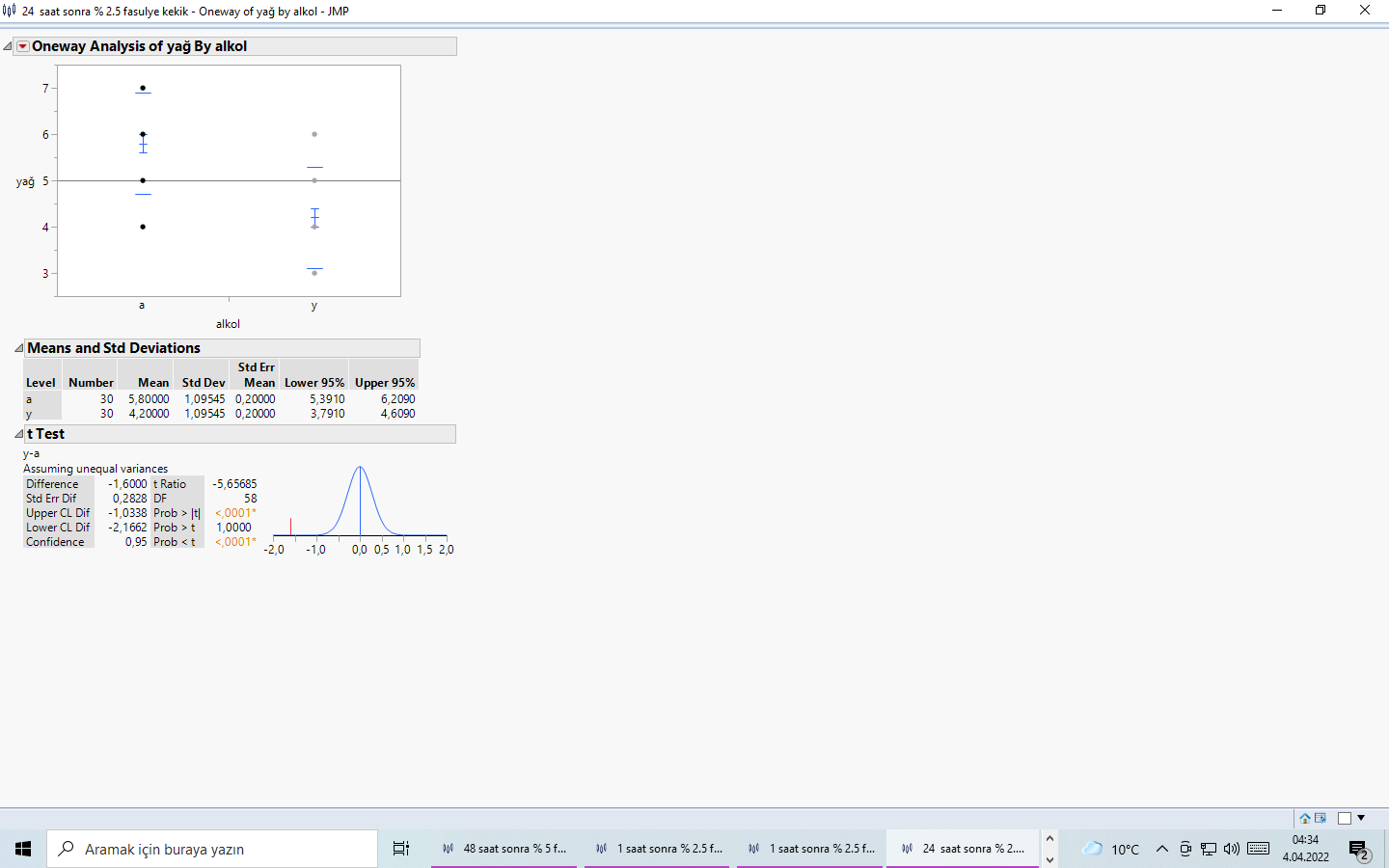


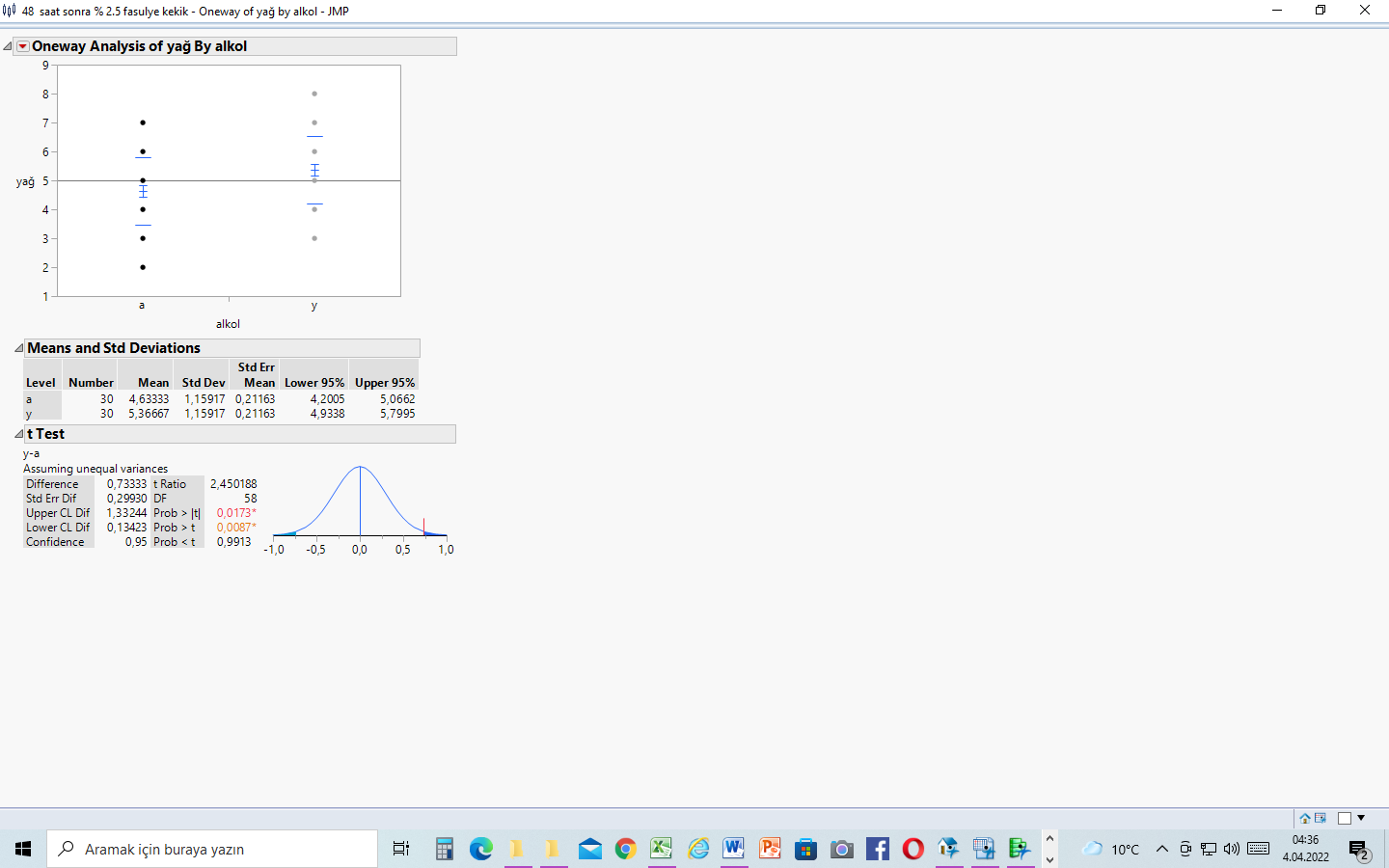


1 saat sonra % 5 fasulye karanfil


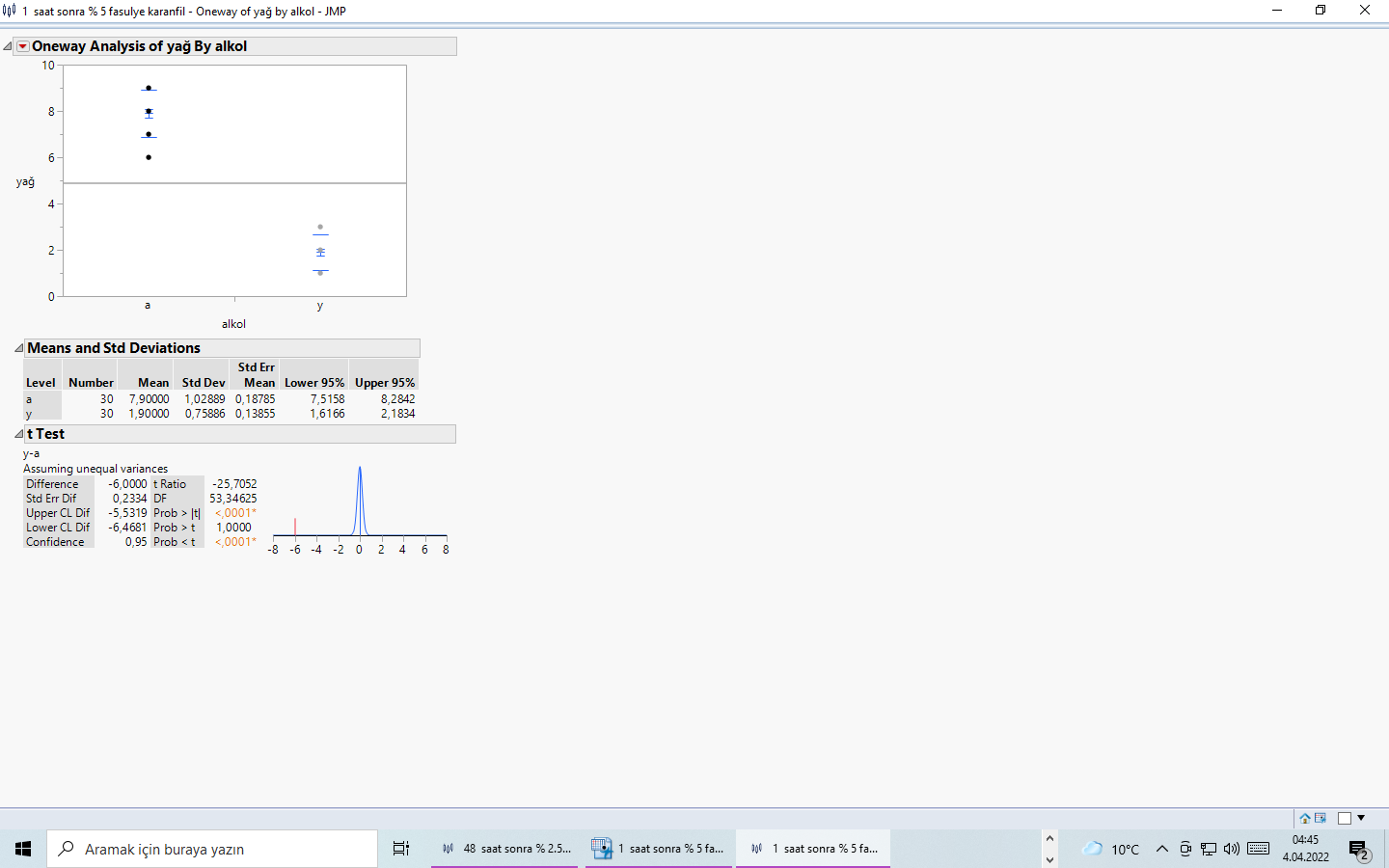


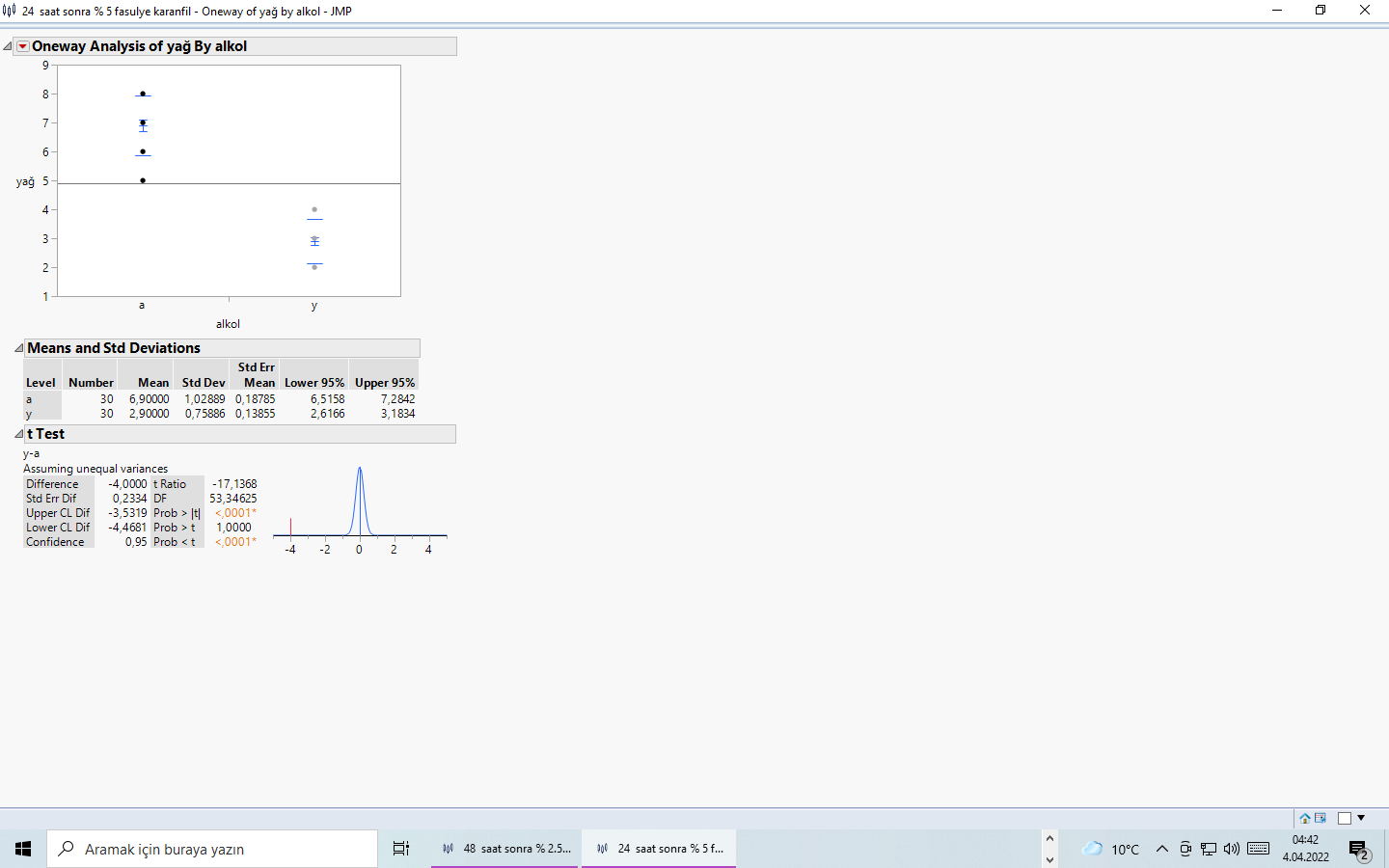


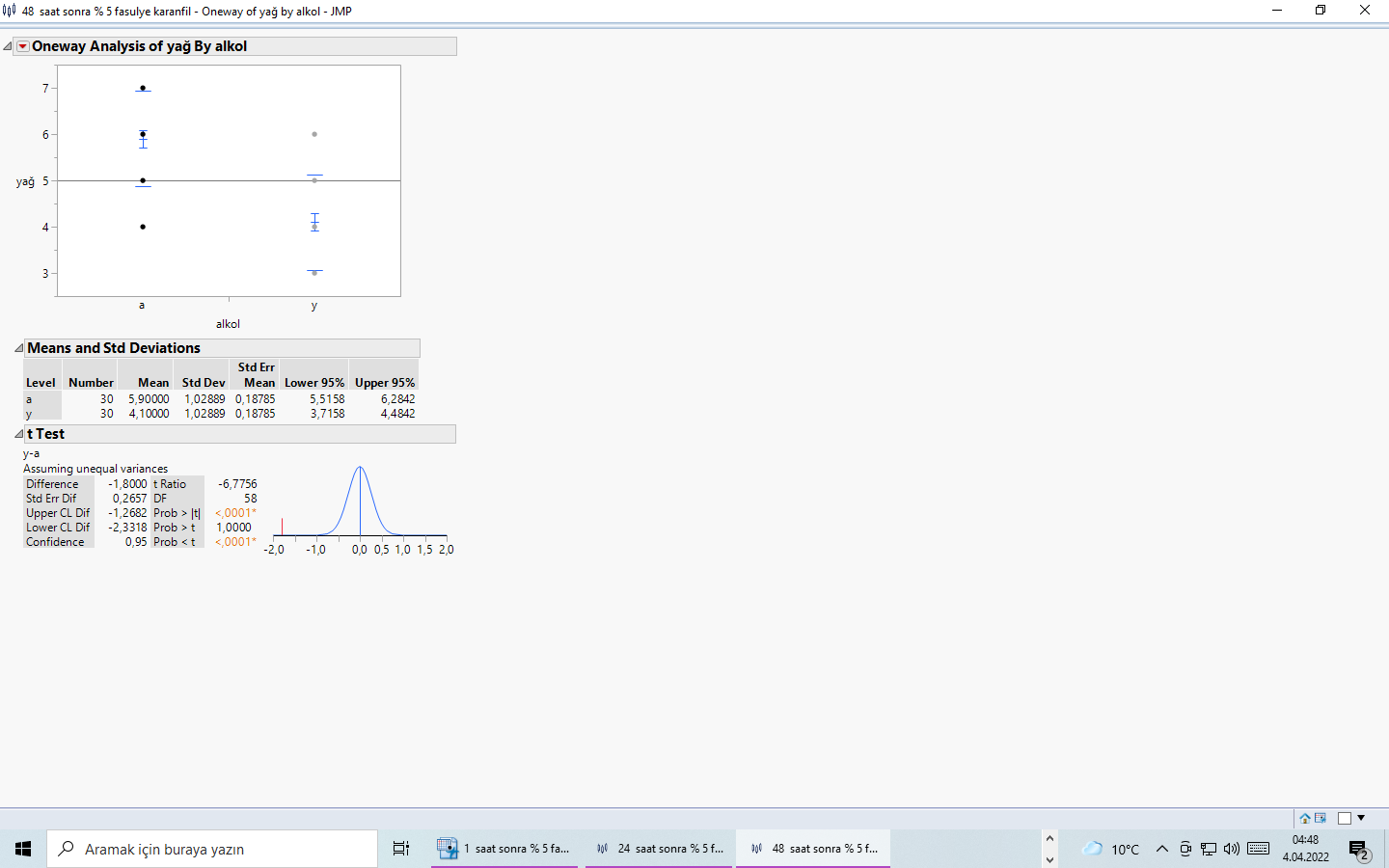


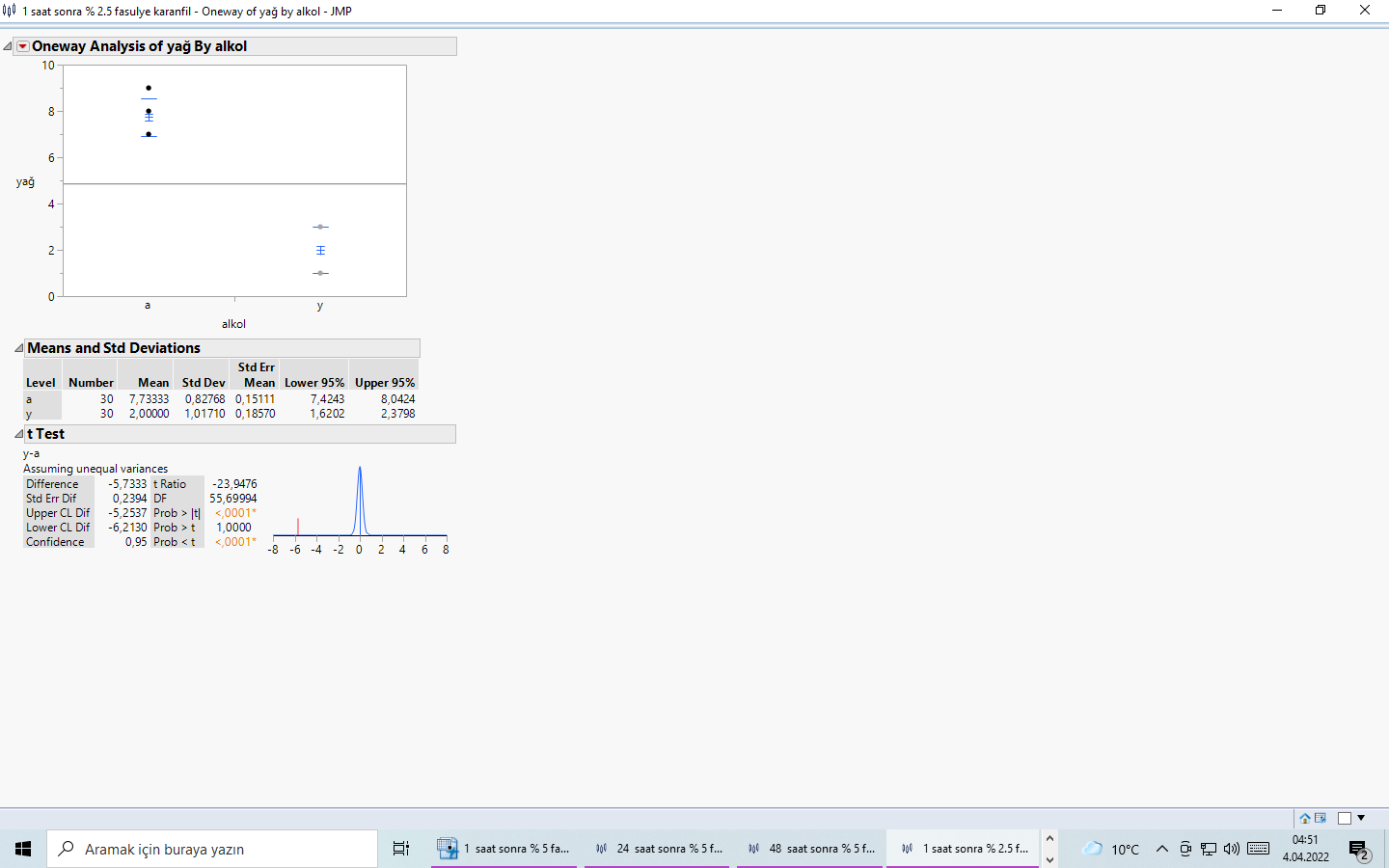


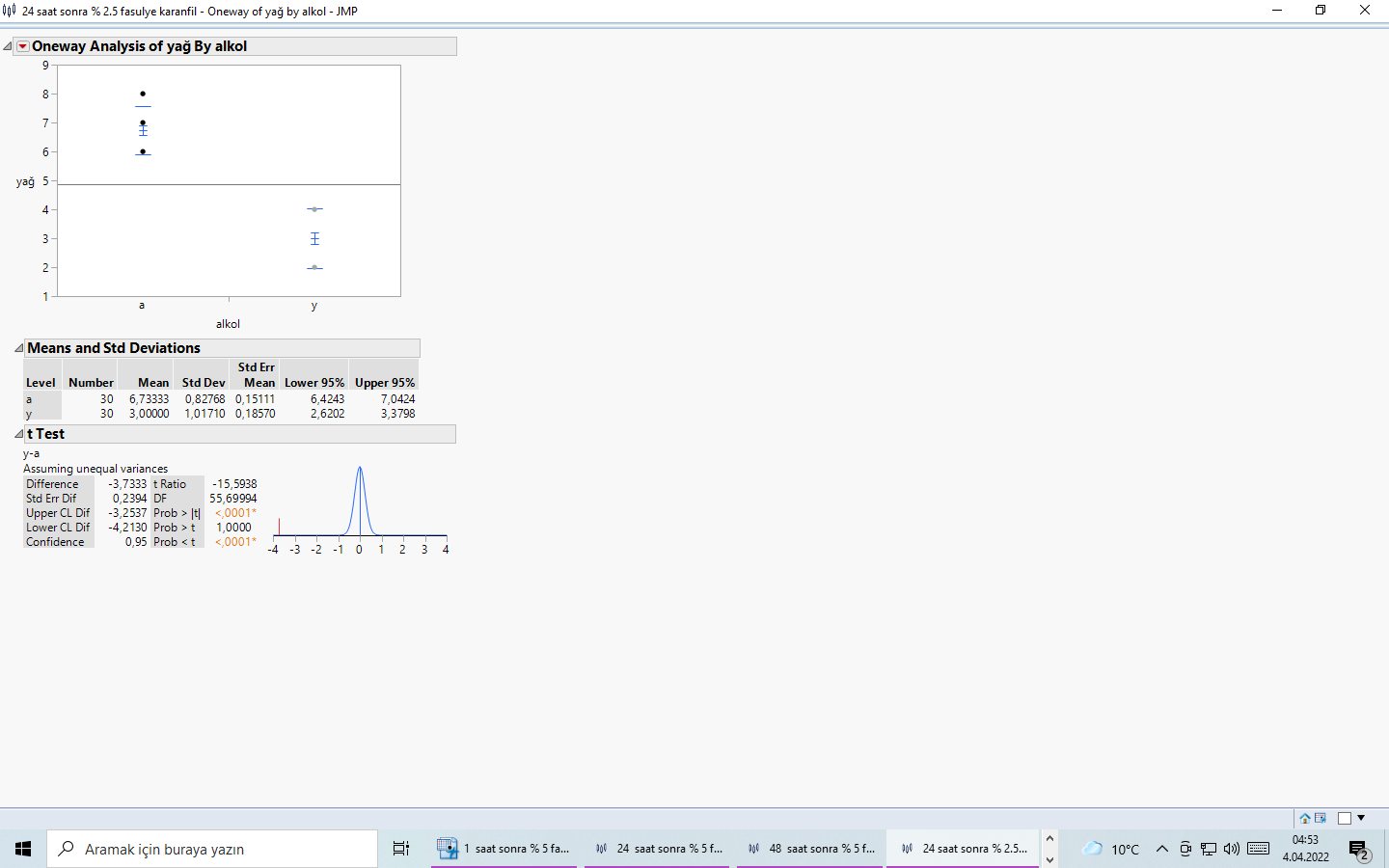


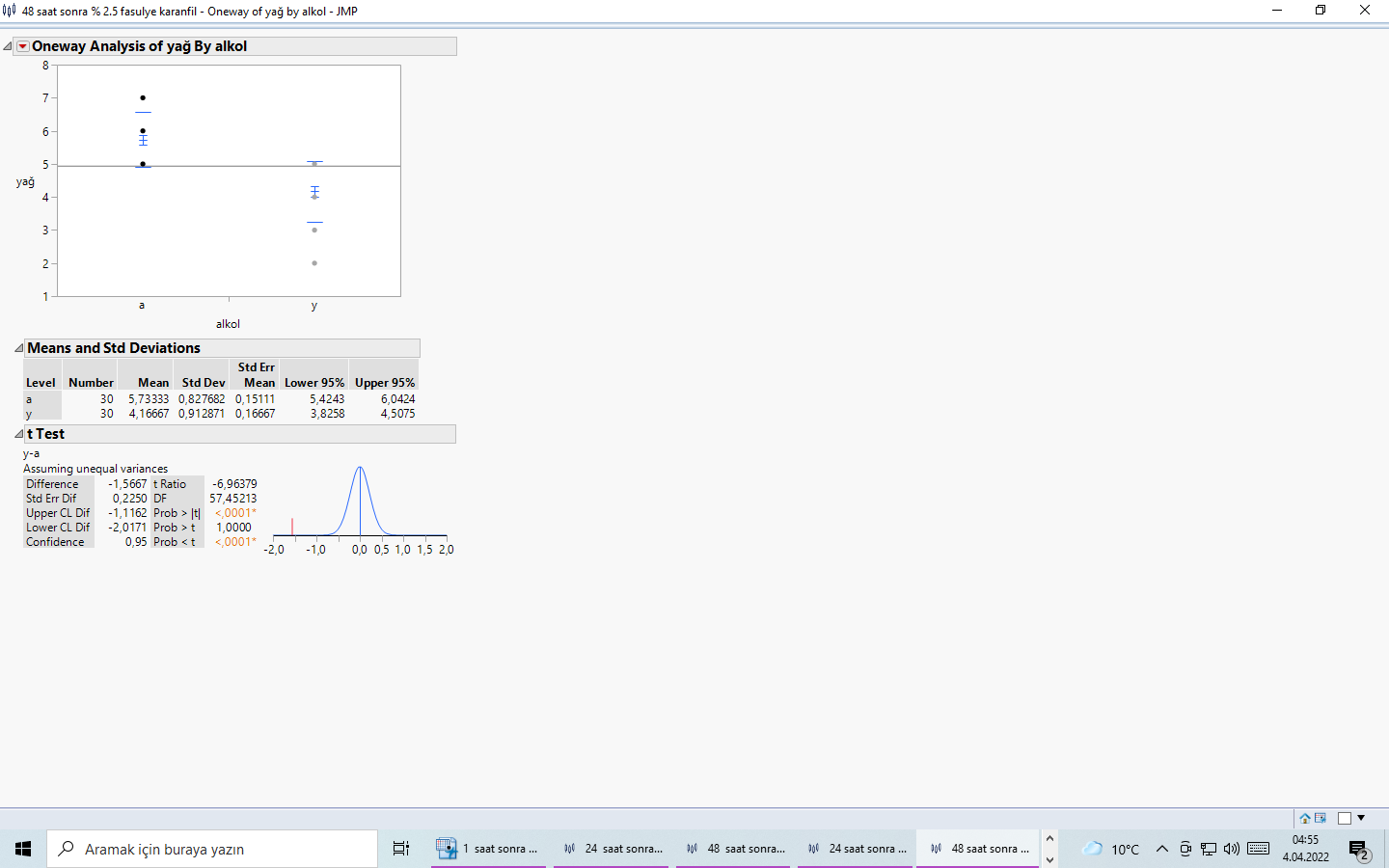


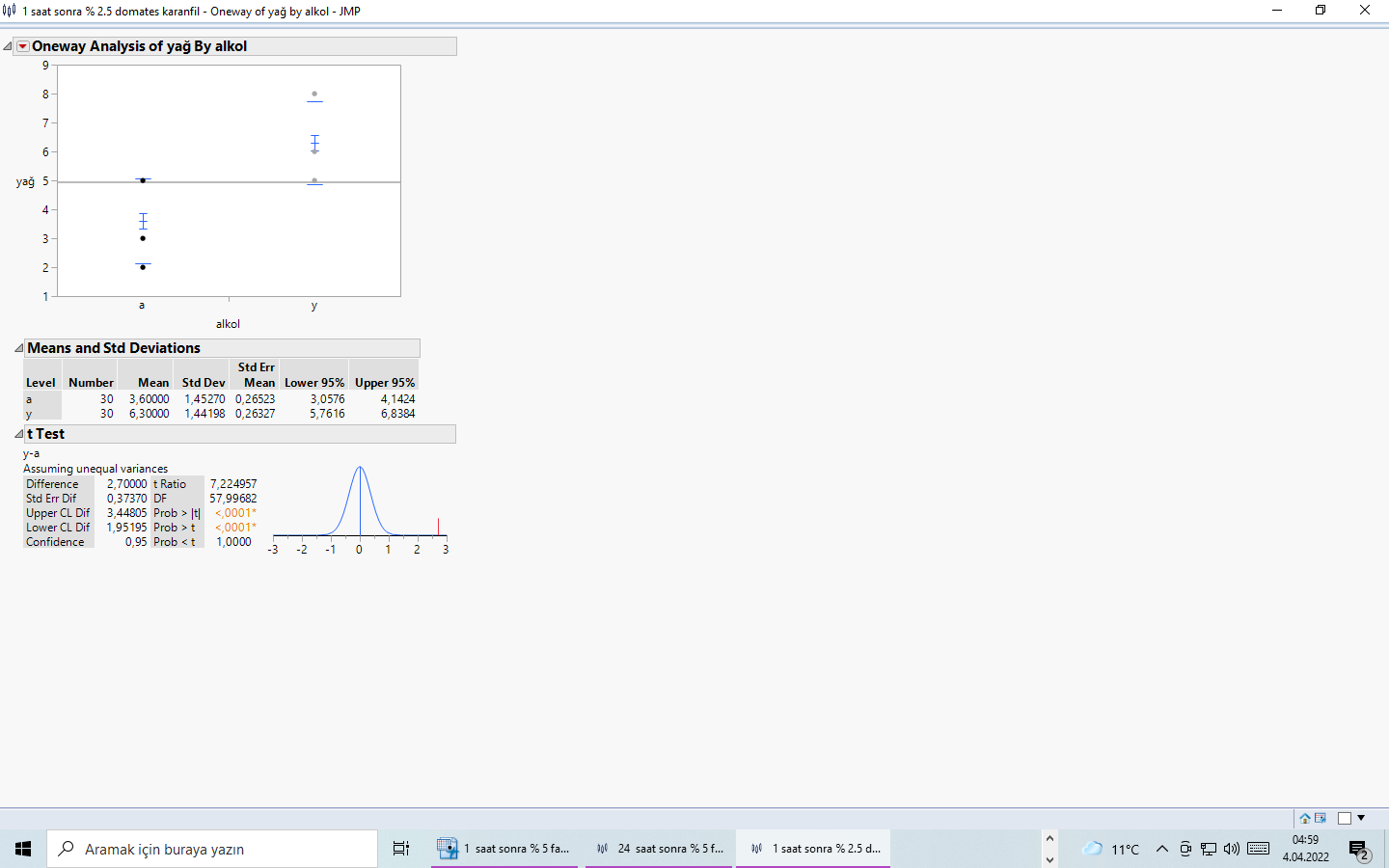


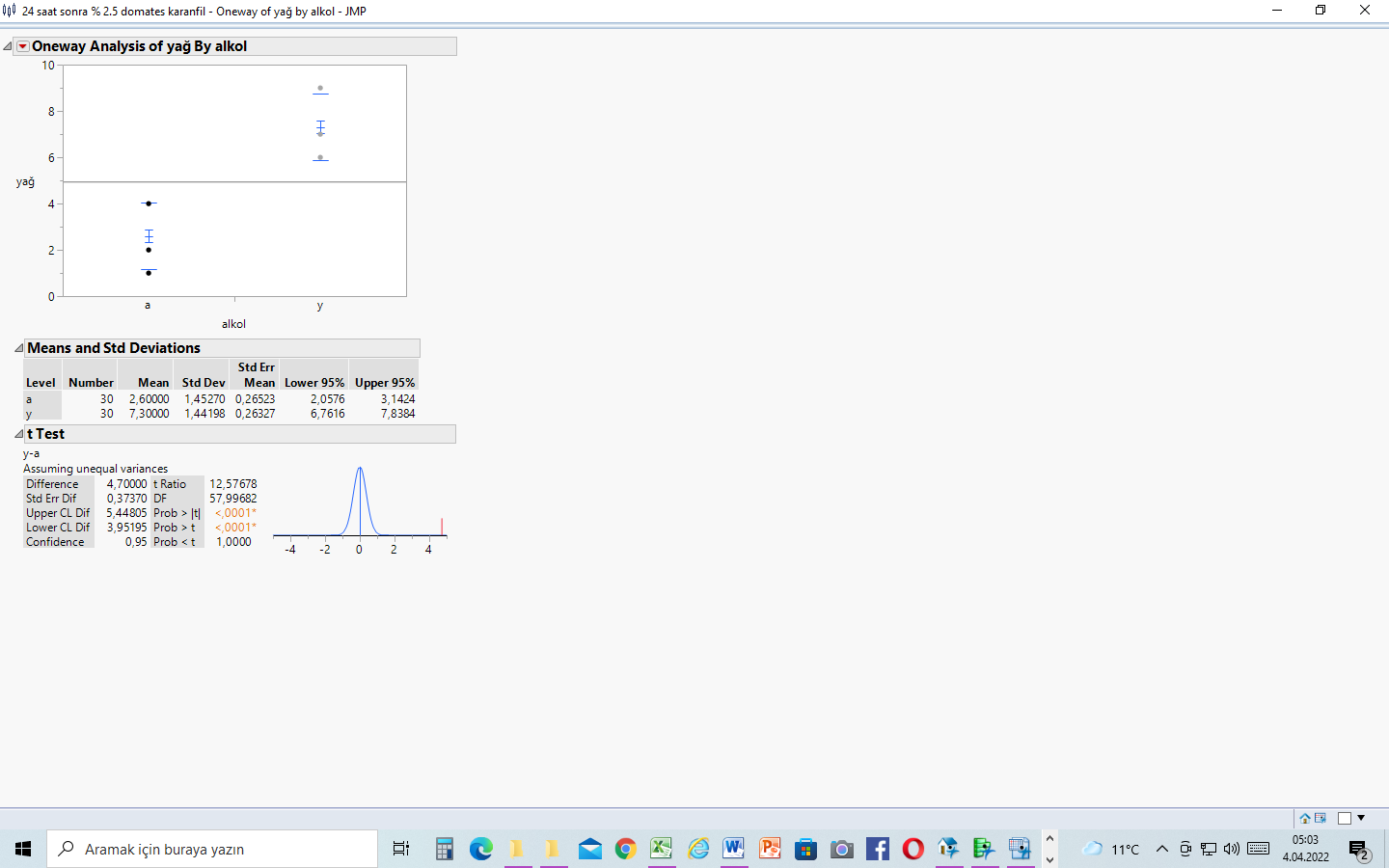


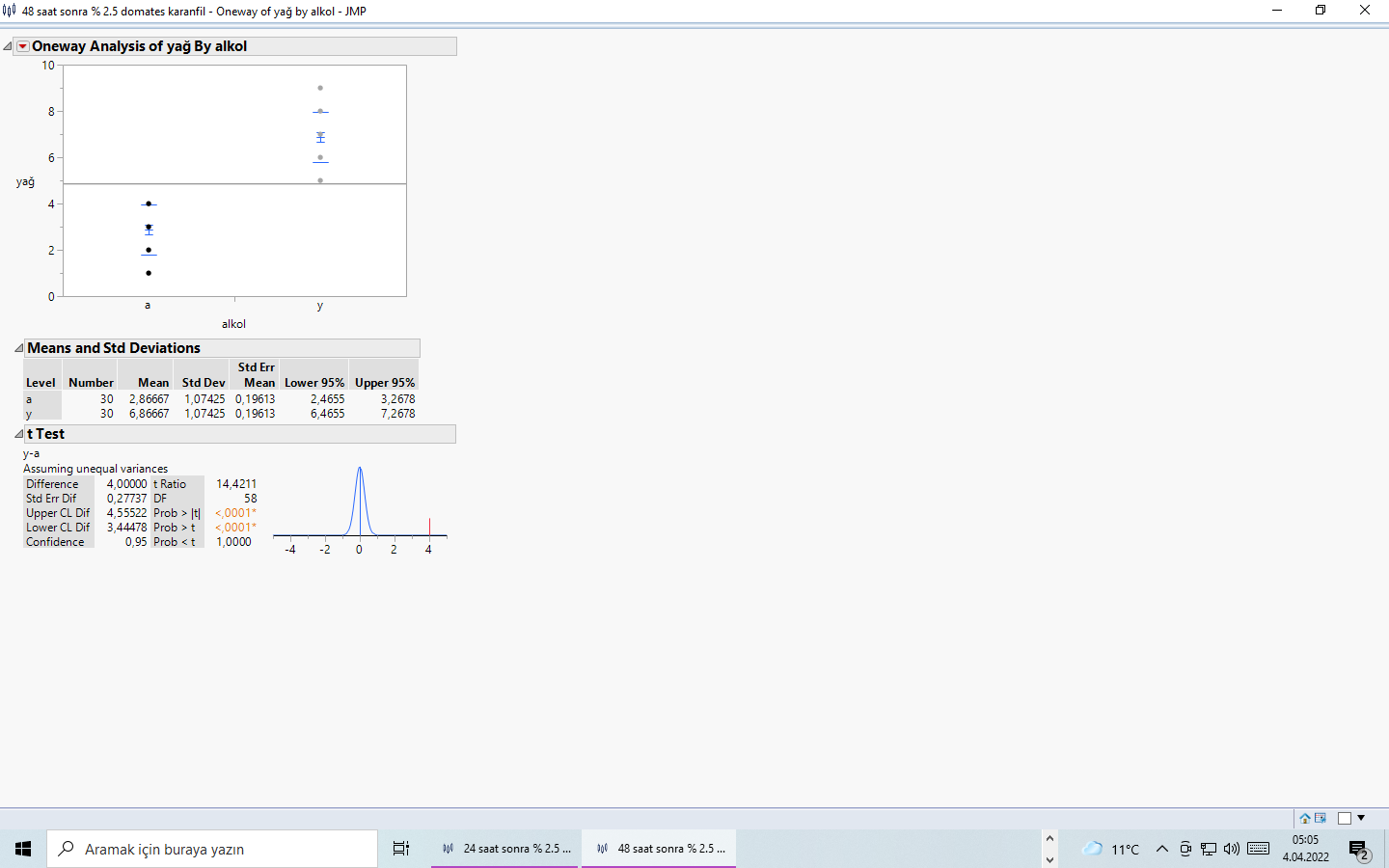


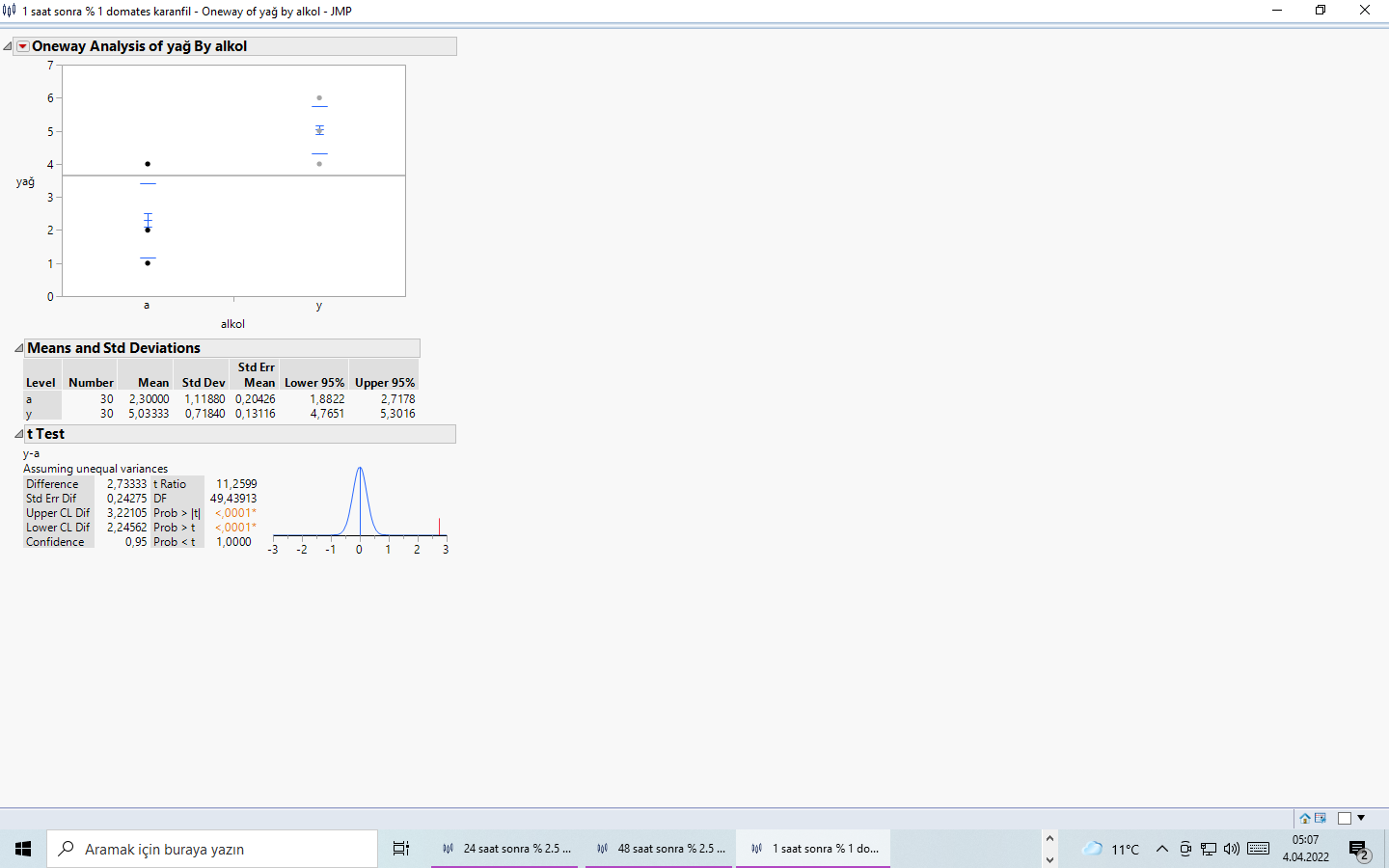


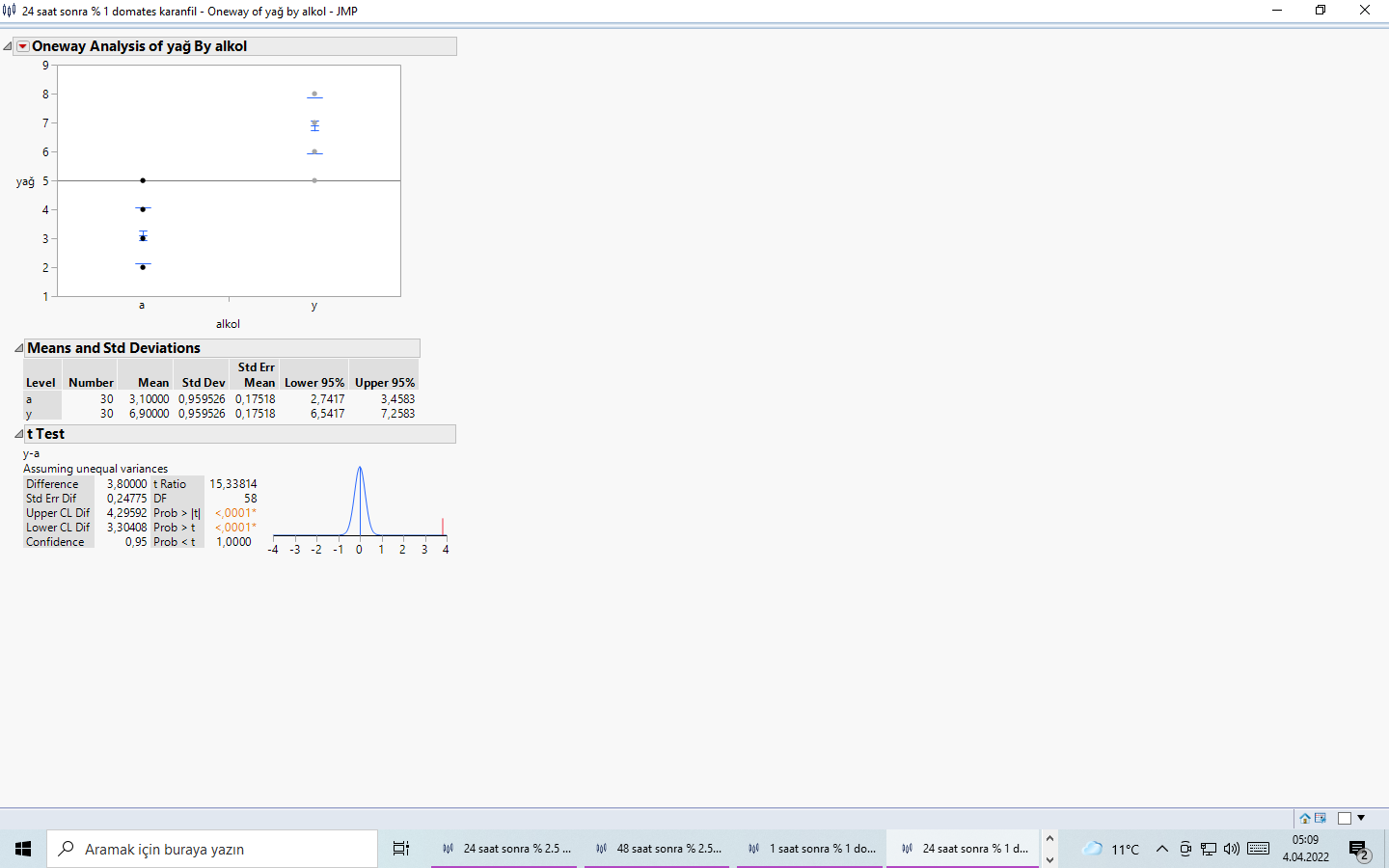


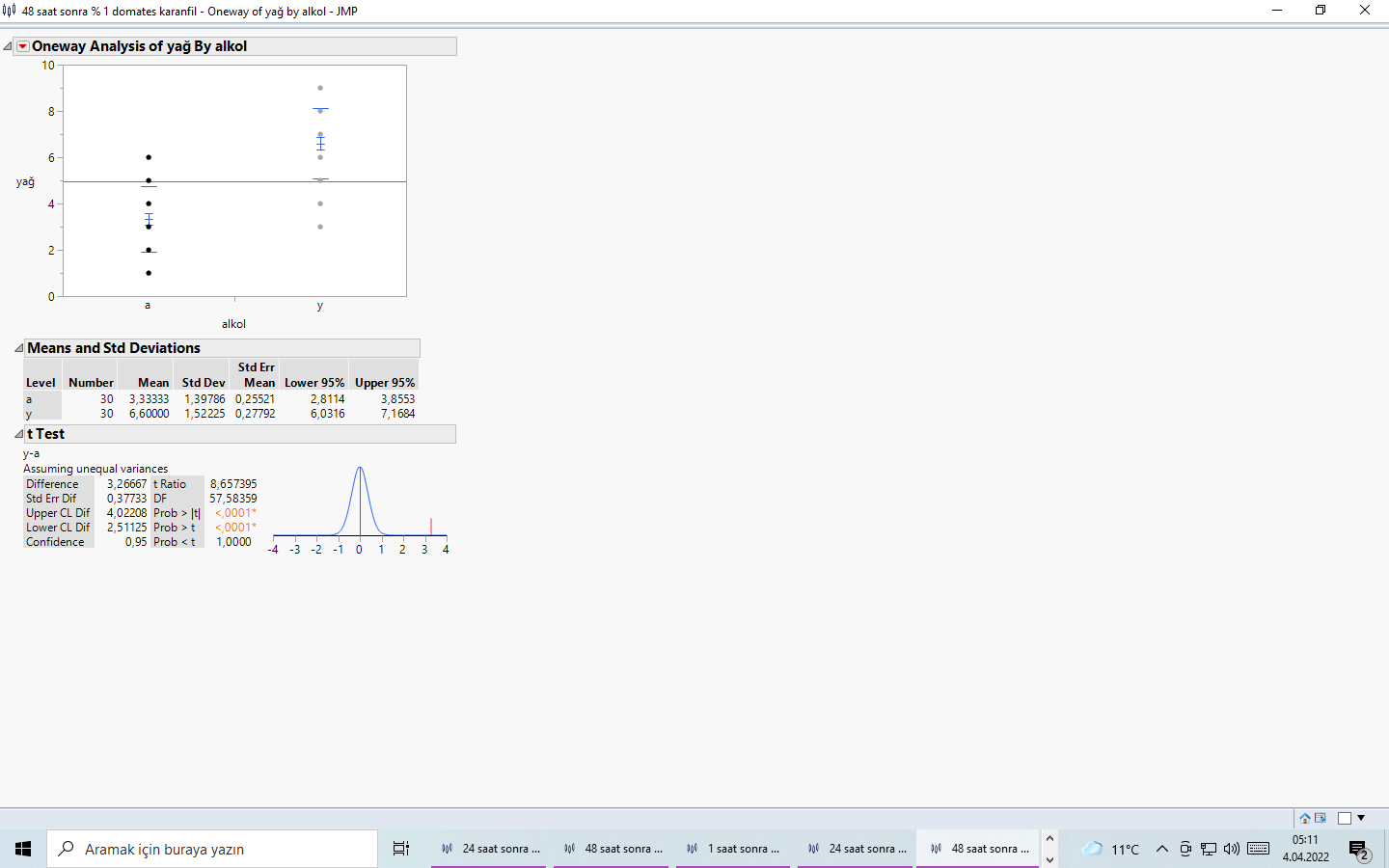


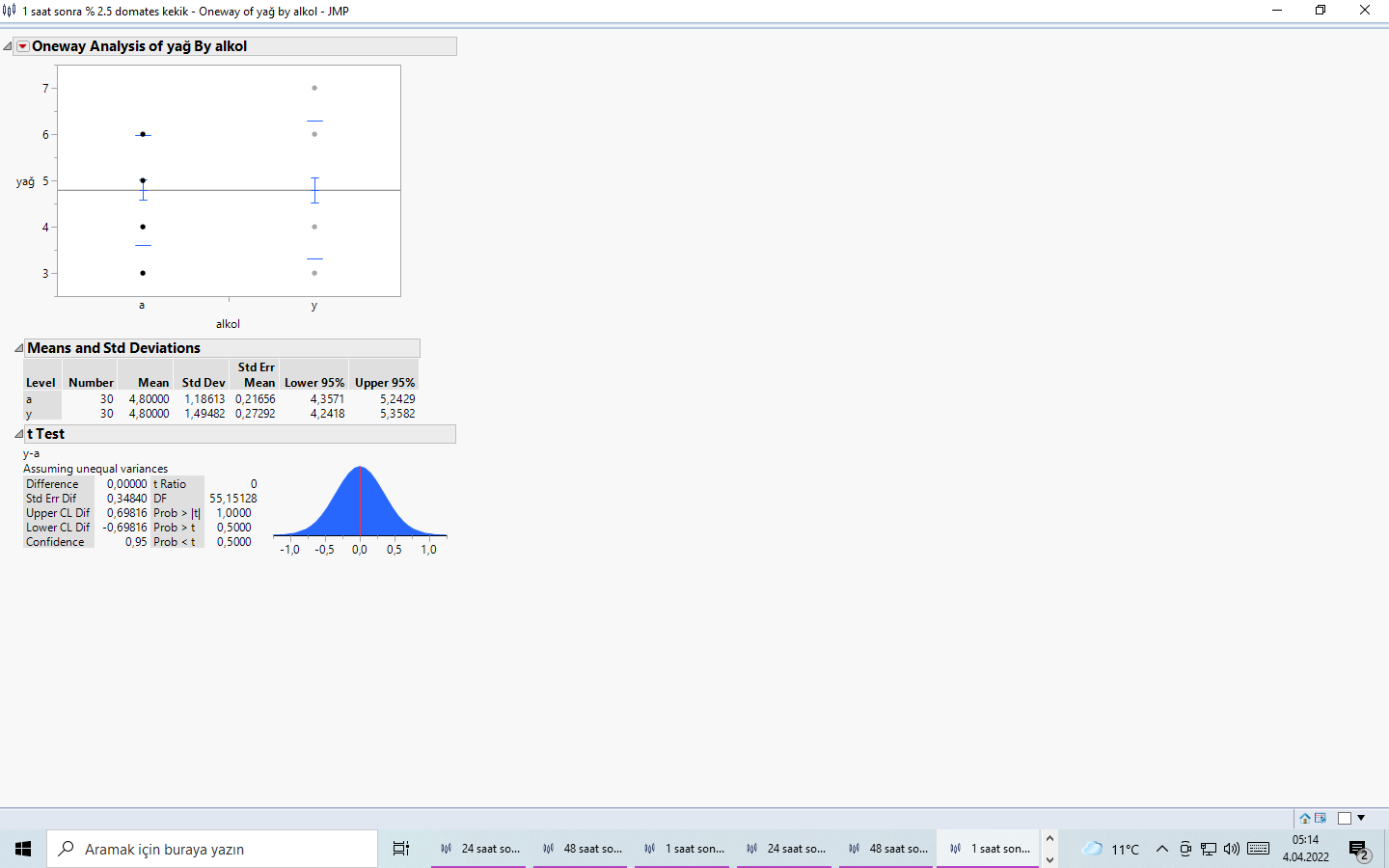


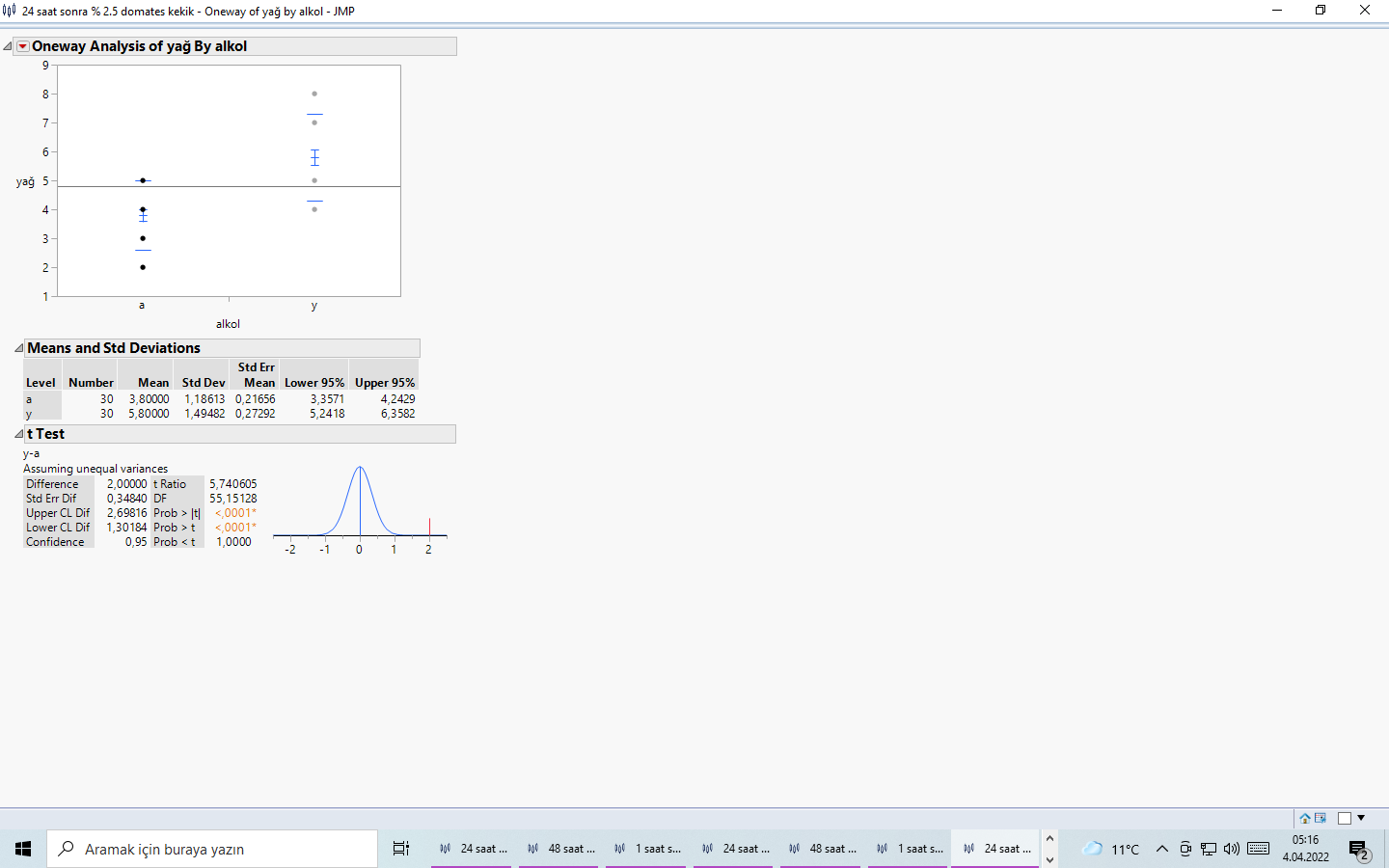


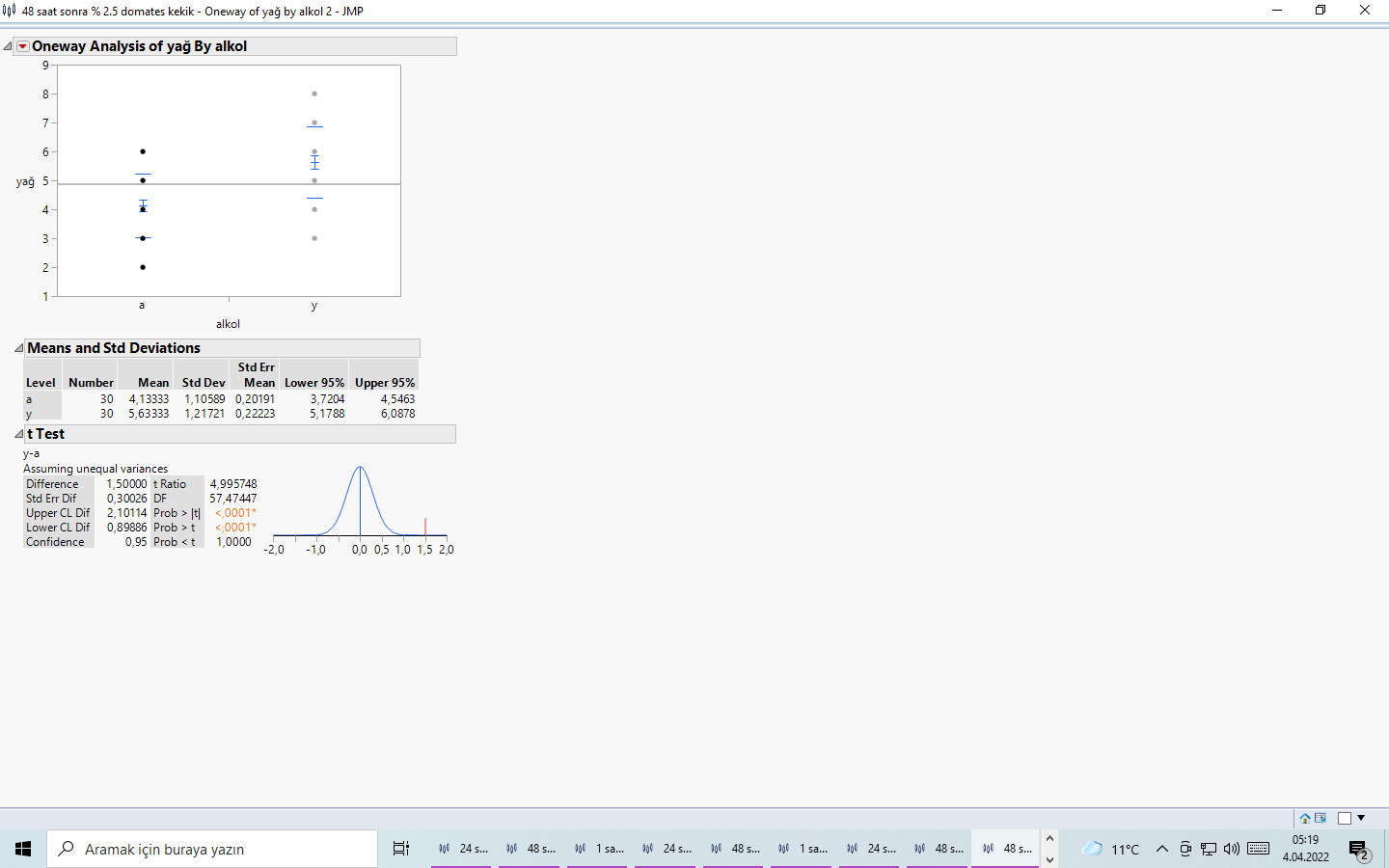


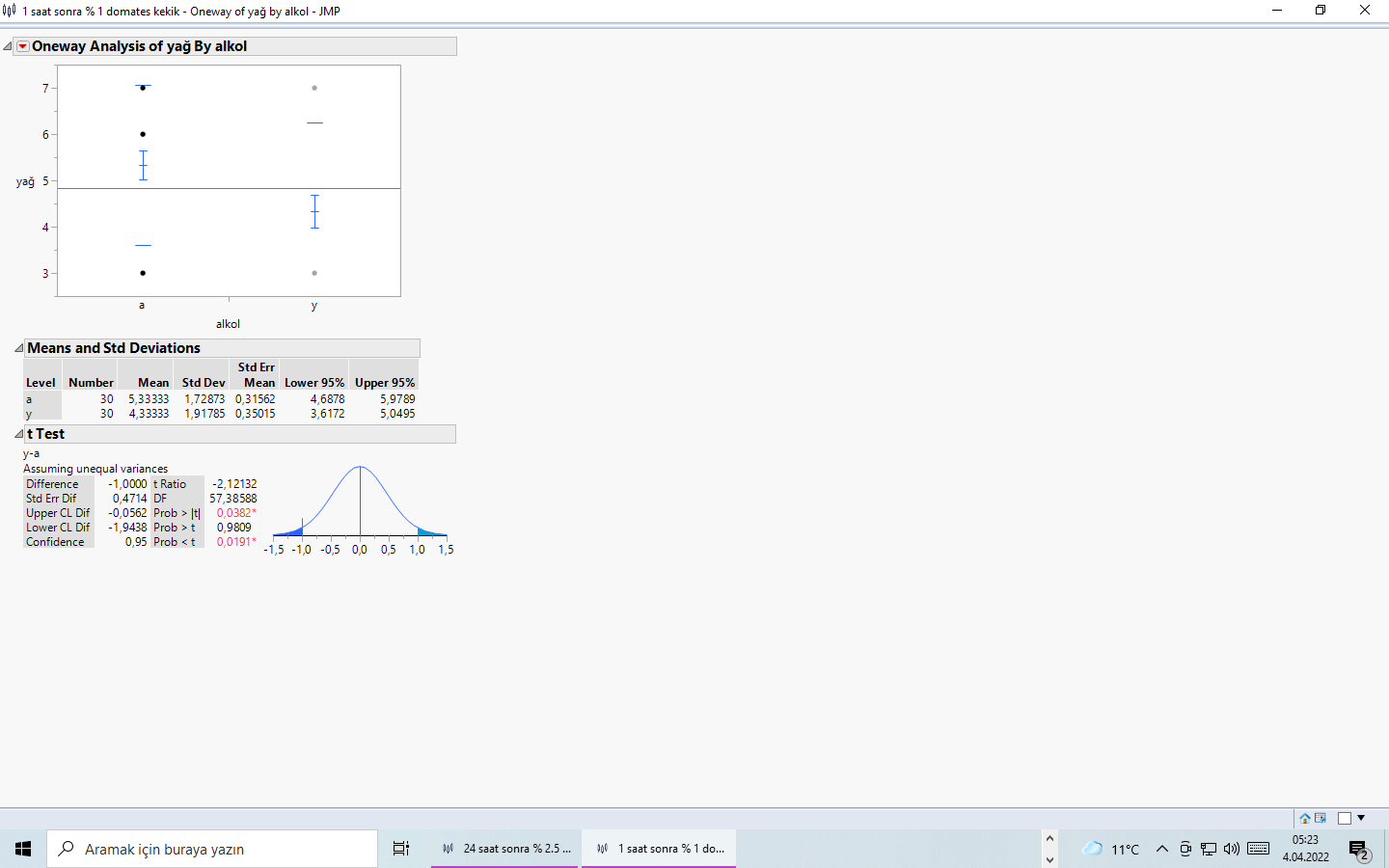


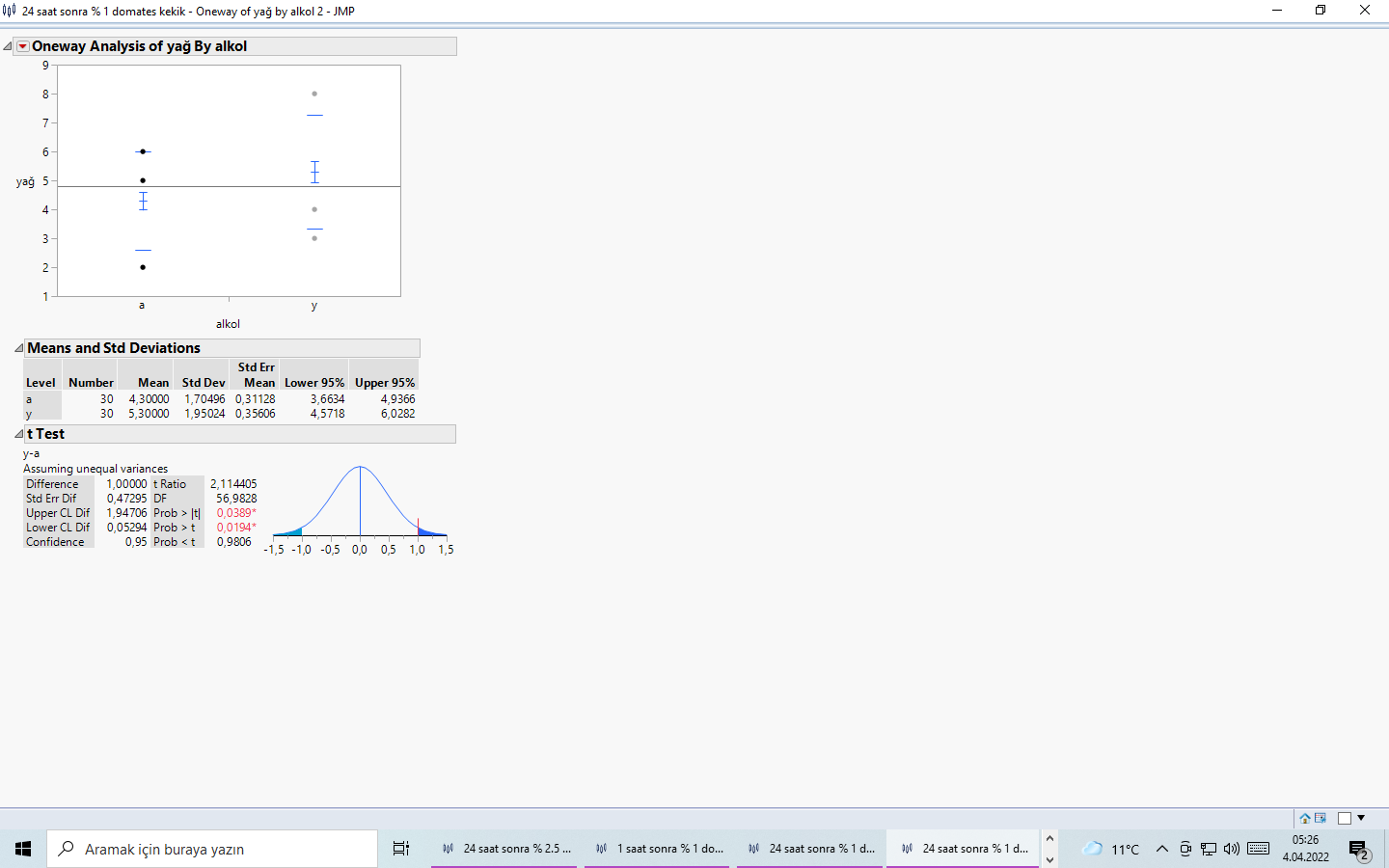


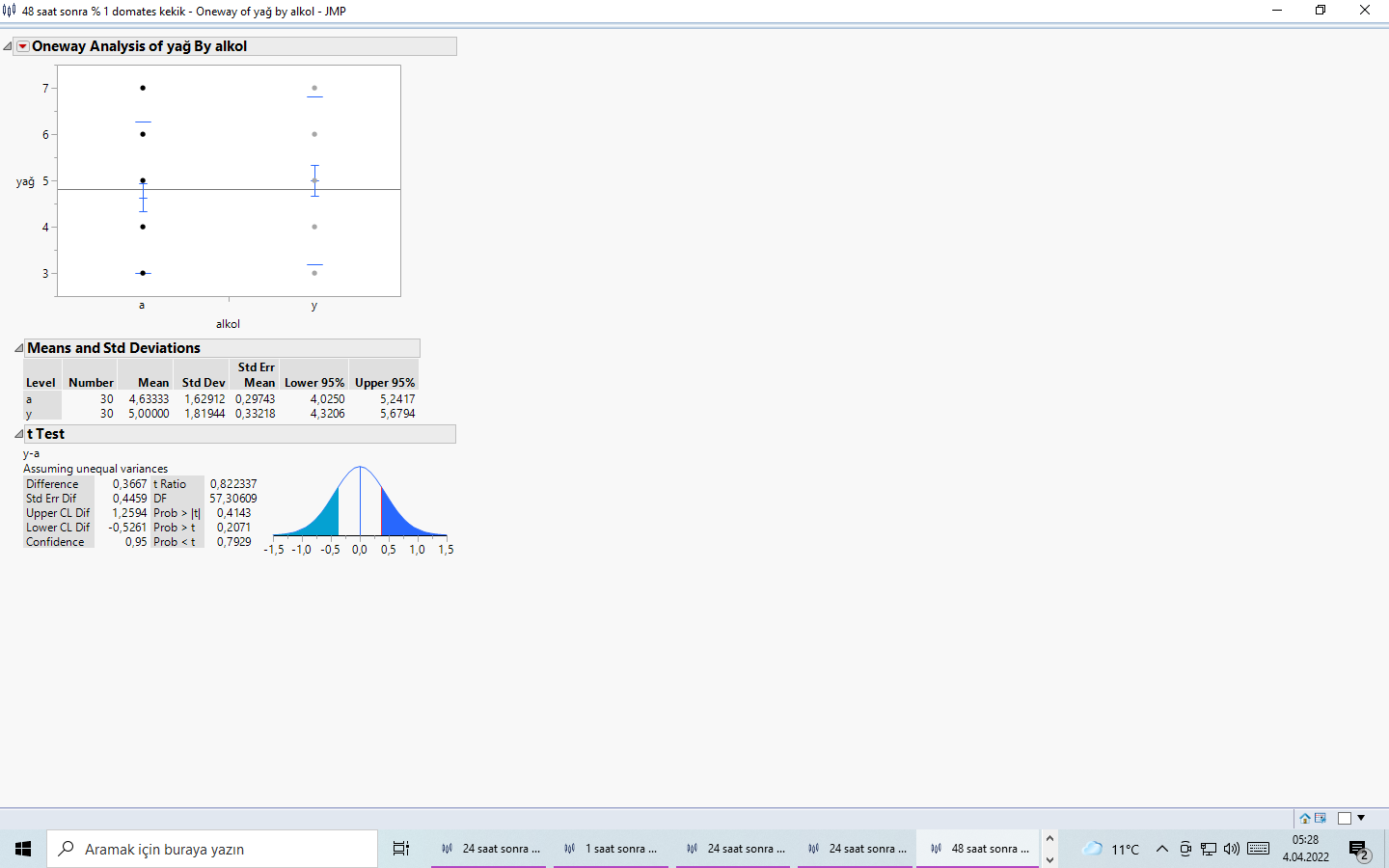

Supplement: Supplemental Information 6 [file peerj-11-14475-s006.docx]
